# Targeted Therapies for Each Subtype of Breast Cancer

**DOI:** 10.1002/mco2.70733

**Published:** 2026-04-12

**Authors:** Aiyu Liu, Puchao Peng, Yeke Zhu, Qiuwen Fei, Weiwei Liu, Shizhen Zhang

**Affiliations:** ^1^ Department of Anesthesiology The First Affiliated Hospital Zhejiang University School of Medicine Hangzhou China; ^2^ Department of Breast Surgery Huzhou Maternity and Child Health Care Hospital Huzhou China; ^3^ Cancer Institute and Key Laboratory of Cancer Prevention and Intervention Ministry of Education the Second Affiliated Hospital Zhejiang University School of Medicine Hangzhou China; ^4^ Department of Breast Surgery and Oncology Key Laboratory of Cancer Prevention and Intervention Ministry of Education The Second Affiliated Hospital Zhejiang University School of Medicine Hangzhou China; ^5^ Department of Laboratory Medicine Zhejiang University School of Medicine Second Affiliated Hospital Hangzhou China

**Keywords:** breast cancer, estrogen receptor targeted therapy, human epidermal growth factor receptor 2, progesterone receptor, triple‐negative breast cancer

## Abstract

Breast cancer (BC) is a clinically heterogeneous malignancy and a leading cause of cancer‐related mortality in women worldwide. It is classified into hormone receptor (HR)‐positive, human epidermal growth factor receptor 2 (HER2)‐positive, and triple‐negative (TNBC) subtypes based on molecular biomarkers. This heterogeneity drives distinct disease progression and treatment responses, making subtype‐specific precision therapy indispensable for improving patient outcomes. While estrogen receptor (ER)‐targeting agents and anti‐HER2 therapies have achieved notable successes, critical challenges remain, including drug resistance, inadequate biomarkers, and limited therapeutic targets for TNBC. This review comprehensively summarizes recent advances in targeted therapies for major BC subtypes: endocrine therapy combined with cyclin‐dependent kinase 4/6 (CDK4/6) or phosphatidylinositol 3‐kinase (PI3K)/protein kinase B (AKT)/mammalian target of rapamycin (mTOR) inhibitors for HR‐positive BC; novel antibody‒drug conjugates (ADCs) such as trastuzumab deruxtecan (T‐DXd) and tyrosine kinase inhibitors (TKIs) for HER2‐positive BC; and trophoblast cell‐surface antigen 2 (Trop‐2) ADCs, immunotherapies, and poly‐ADP‐ribose polymerase (PARP) inhibitors for TNBC. It also discusses cross‐subtype therapeutic platforms (ADCs, PI3K/AKT/mTOR pathway) and emerging modalities (chimeric antigen receptor [CAR] T‐cell therapy, proteolysis‐targeting chimeras [PROTACs]). By analyzing successes, challenges, and translational potential, this review provides a clear framework for clinicians and researchers, advancing personalized treatment optimization and addressing unmet clinical needs in BC precision oncology.

## Graphical abstract

This review systematically summarizes the advances in targeted therapies for the three major molecular subtypes of breast cancer (BC), highlighting key strategies, agents, and clinical progress to guide personalized treatment. HR‐positive (luminal) BC: Dominated by estrogen receptor (ER) signaling, with endocrine therapies as the cornerstone—including selective estrogen receptor modulators (SERMs; e.g., tamoxifen), aromatase inhibitors (AIs; e.g., anastrozole) for postmenopausal patients, and selective estrogen receptor degraders (SERDs; e.g., fulvestrant). Novel oral SERDs (e.g., elacestrant) and proteolysis‐targeting chimera (PROTAC) ER degraders (vepdegestrant) address endocrine resistance. In addition, CDK4/6 inhibitors (palbociclib and ribociclib) and PI3K/AKT/mTOR pathway inhibitors (everolimus, alpelisib, and capivasertib) synergize with endocrine therapy to enhance efficacy, particularly in advanced disease. HER2‐positive BC: Transformed by HER2‐targeted agents, starting with trastuzumab (monoclonal antibody) and expanding to dual blockade (trastuzumab + pertuzumab). Tyrosine kinase inhibitors (TKIs; e.g., lapatinib and tucatinib) overcome resistance and penetrate the blood‒brain barrier, targeting CNS metastases. Antibody‒drug conjugates (ADCs) such as T‐DM1 and trastuzumab deruxtecan (T‐DXd) show superior efficacy, with T‐DXd extending benefits to HER2‐low BC. Triple‐negative BC (TNBC): Emerging targets include Trop‐2‐directed ADCs (sacituzumab govitecan, sacituzumab tirumotecan) for advanced disease. PARP inhibitors (olaparib and talazoparib) benefit *BRCA*‐mutated TNBC. Immunotherapy (pembrolizumab and toripalimab) and CAR T‐cell therapy (targeting MUC1 and EGFR) show promise, leveraging TNBC immunogenicity. Other strategies target VEGF, EGFR, and gpNMB. Collectively, these advances underscore BC as a model for precision oncology, with ongoing efforts to refine biomarkers and develop novel agents to address unmet needs, paving the way for optimized personalized therapies. The figure was partially created with the assistance of the BioRender tool.

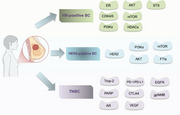

## 1 Introduction

**FIGURE 1:**
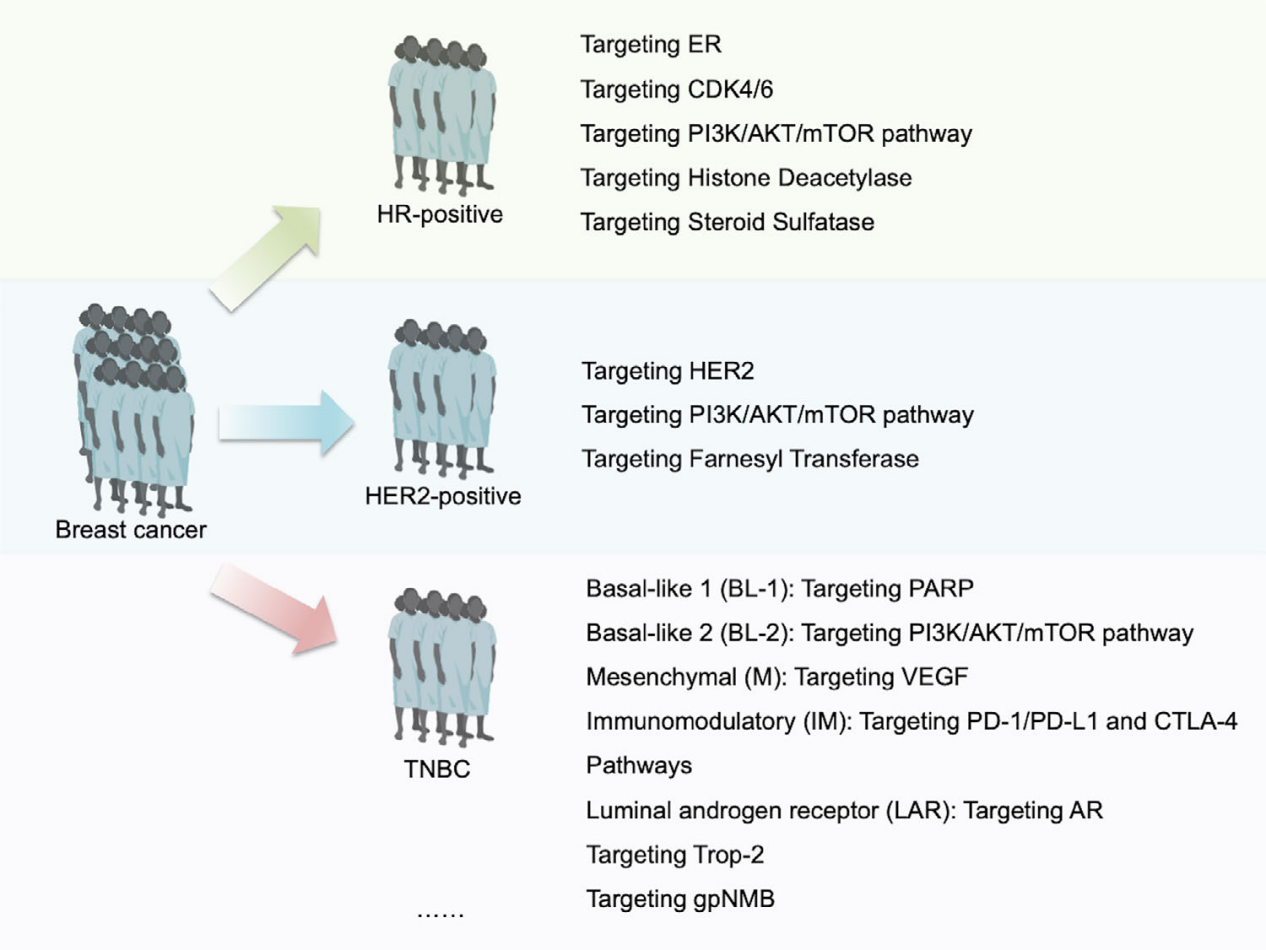
Schematic of core therapeutic targets across major breast cancer subtypes. Illustrates key molecular targets (ER, HER2, PARP, etc.) corresponding to HR‐positive (Luminal), HER2‐positive, and triple‐negative (TNBC) breast cancer, aligned with subtype‐specific oncogenic drivers. Partially created with the assistance of the BioRender tool.

Breast cancer (BC) is the most frequently diagnosed malignancy and the second leading cause of cancer‐related mortality among women worldwide, imposing a substantial global health burden [1, 2]. A defining feature of BC is its profound clinical and biological heterogeneity—tumors exhibit distinct morphological, molecular, and behavioral profiles that directly influence disease progression, treatment response, and patient outcomes [3]. Clinically, BC is stratified into three principal molecular subtypes based on biomarker expression: hormone receptor‐positive (HR‐positive, encompassing estrogen receptor [ER] and progesterone receptor [PR], often referred to as luminal BC), human epidermal growth factor receptor 2‐enriched (HER2‐positive), and triple‐negative breast cancer (TNBC; lacking ER, PR, and HER2 expression). Luminal BC constitutes the majority of BC cases (approximately 60%–70%), characterized by dependence on the estrogen/ER signaling pathway for growth and benefiting from endocrine therapy [4]. HER2‐positive BC accounts for 15%–20% of all BC cases and was once considered one of the most aggressive subtypes, with HER2 amplification/overexpression driving malignant behaviors and significantly poor survival [5], but has seen drastically improved outcomes with anti‐HER2 agents such as trastuzumab [6, 7, 8]. TNBC represents 15%–20% of BC cases and remains the most challenging subtype due to the absence of targetable ER, PR, or HER2 receptors [9, 10]. Molecular profiling has further subdivided TNBC into six intrinsic subtypes (basal‐like 1/2, mesenchymal, immunomodulatory (IM), mesenchymal stem‐like (MSL), and luminal androgen receptor [LAR]), each with unique biological drivers, but targeted therapies for TNBC remained limited until recent years, with chemotherapy as the mainstay until the advent of poly‐ADP‐ribose polymerase (PARP) inhibitors (for *BRCA*‐mutated cases) and immune checkpoint inhibitors [11, 12, 13]. Each subtype exhibits distinct biological behaviors that critically inform tumor progression patterns and guide targeted therapeutic selection (Figure 1) [14, 15].

Precision medicine in BC is anchored by the identification of actionable biomarkers, such as ER/PR for endocrine therapy and HER2 for anti‐HER2 agents [2, 16]. Over the past quarter‐century, this approach has established BC as a model of precision oncology. The management of HR‐positive disease has evolved from single‐agent endocrine therapy to combination regimens with cyclin‐dependent kinase 4/6 (CDK4/6) or PI3K/AKT/mTOR pathway inhibitors. Similarly, HER2‐positive BC treatment has advanced from trastuzumab monotherapy to multi‐agent strategies that now include pertuzumab, tyrosine kinase inhibitors (TKIs), and ADCs [17, 18]. Even TNBC, once a “targetless” subtype, now has approved therapies targeting Trop‐2, PARP, or immune checkpoints, underscoring the power of precision medicine [13].

**FIGURE 2:**
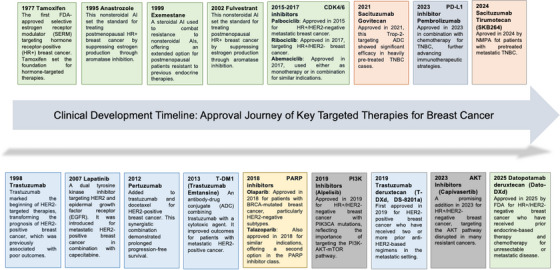
Timeline of clinical development for key targeted therapies in breast cancer. Summarizes the chronological approval and landmark clinical advances of core agents, including endocrine therapies, anti‐HER2 drugs, ADCs, and immunotherapies.

The clinical development timeline of key targeted therapies for BC is shown in Figure 2. The primary objective of this review is to provide a comprehensive, evidence‐based analysis of current and investigational targeted therapies for the major immunophenotypes of BC. First, we focus on luminal (HR‐positive) BC, detailing therapies targeting the ER pathway (e.g., selective estrogen receptor modulators [SERMs], aromatase inhibitors [AIs], selective estrogen receptor degraders [SERDs]), cell cycle regulators (CDK4/6 inhibitors), and resistance‐associated pathways (PI3K/AKT/mTOR, histone deacetylases [HDACs]), with an emphasis on addressing endocrine resistance. Next, we discuss HER2‐positive BC, covering monoclonal antibodies (mAbs), TKIs, ADCs, and pathway inhibitors (e.g., PI3K/AKT/mTOR inhibitors) that have transformed its treatment, as well as challenges such as brain metastases and acquired resistance. We then address TNBC, exploring emerging targeted approaches (e.g., Trop‐2 ADCs, PARP inhibitors (PARPis), androgen receptor [AR] antagonists) and immunotherapies (PD‐1/PD‐L1 inhibitors, chimeric antigen receptor [CAR] T‐cell therapy) that are overcoming its historical lack of targets. Finally, we synthesize the successes and persistent challenges of BC targeted therapy and outline future translational directions to further optimize personalized treatment. By organizing content around subtype‐specific molecular drivers and treatment strategy evolution, this review aims to provide clinicians and researchers with a clear framework to navigate the complex and rapidly advancing BC targeted therapy landscape.

## 2 Targeting HR‐Positive (Luminal) BC

**FIGURE 3:**
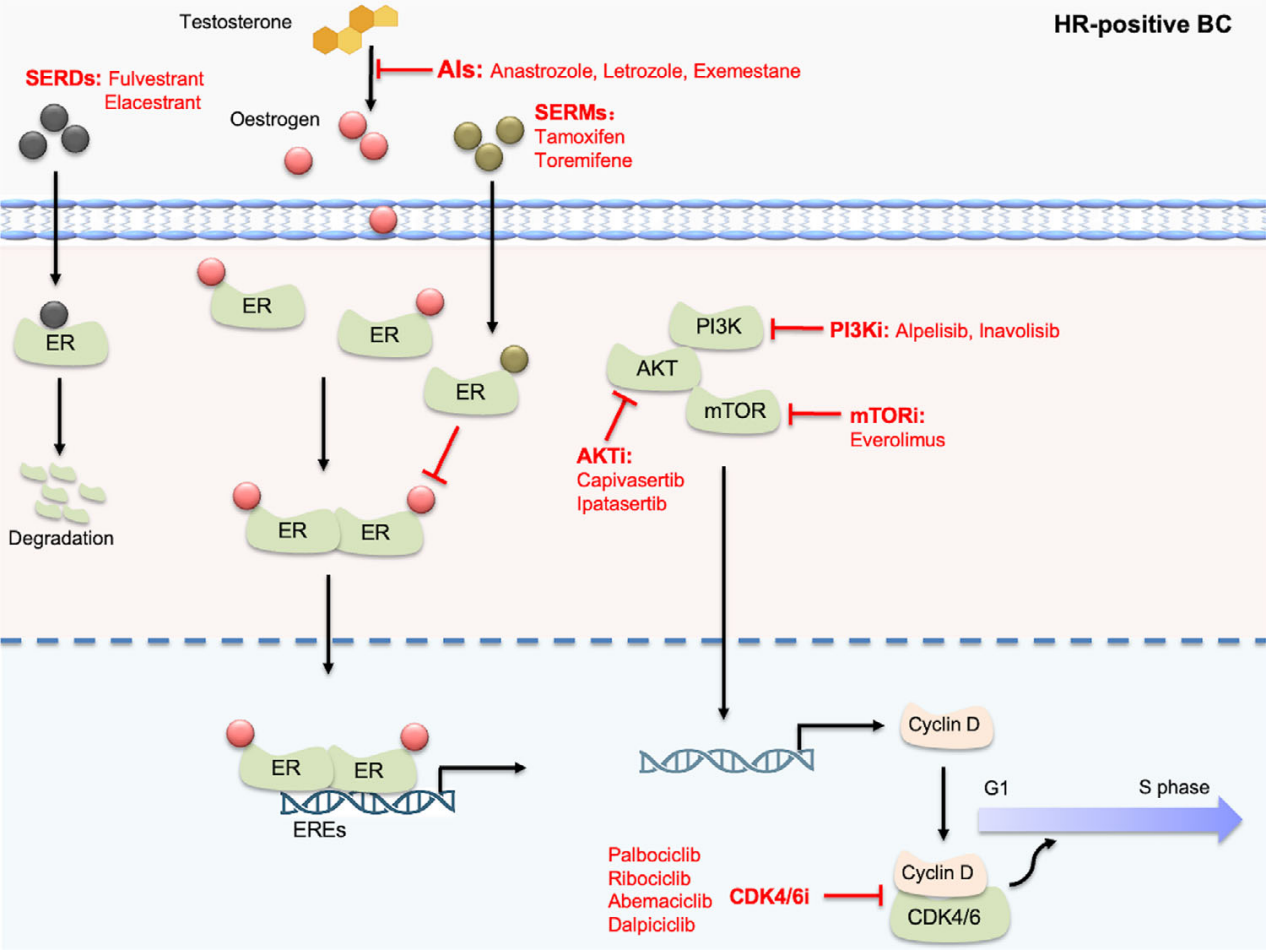
Mechanisms of targeted therapy for HR‐positive (luminal) breast cancer. Highlights the core therapeutic pathways—estrogen receptor (ER) signaling, CDK4/6, and PI3K/AKT/mTOR—for the treatment of hormone receptor (HR)‐positive breast cancer. EREs: estrogen response elements, SERMs: selective estrogen receptor modulators, AIs: aromatase inhibitors, SERDs: selective estrogen receptor degraders.

The growth and progression of luminal BC are predominantly driven by the estrogen/ER signaling pathway, and clinical evidence has established that ER‐positive tumors, particularly those with low Ki‐67 expression, derive limited benefit from conventional chemotherapy compared to ER‐negative tumors. As a result, adjuvant endocrine therapy remains the cornerstone of treatment for this subtype [4], and the emergence of acquired resistance and intra‐subtype heterogeneity has driven the development of novel targeted strategies [19]. This section systematically reviews therapies targeting the core ER pathway, mechanisms to overcome resistance, and emerging approaches for this subtype (Figure 3).

### 2.1 Cornerstone of Therapy: Targeting the ER

Endocrine therapy (hormone therapy) serves as the cornerstone of treatment for HR‐positive BC, offering a targeted and often better‐tolerated first‐line alternative to chemotherapy. By directly interfering with ER signaling, this approach effectively suppresses tumor growth in ER‐dependent cancers. The three primary classes of endocrine agents, SERMs, AIs, and SERDs, each employ distinct mechanisms to block estrogen‐driven proliferation [20].

#### 2.1.1 SERMs

SERMs (e.g., tamoxifen and toremifene) act as tissue‐specific modulators, antagonizing ER in breast tissue while exerting estrogenic effects in other organs (e.g., bones and uterus) [17]. This dual activity makes SERMs particularly valuable for premenopausal patients, where they remain a first‐line adjuvant therapy option. For example, tamoxifen reduces the risk of recurrence by ∼50% in premenopausal ER‐positive early BC and improves OS in both early and metastatic settings [21]. Notably, SERMs are also used in premenopausal patients receiving ovarian suppression (e.g., with gonadotropin‐releasing hormone agonists), where they complement estrogen reduction to enhance therapeutic efficacy [22].

#### 2.1.2 AIs

AIs (anastrozole, letrozole, and exemestane) have become the first‐line choice for postmenopausal women by drastically reducing circulating estrogen levels through irreversible inhibition of the aromatase enzyme [23]. Extensive clinical evidence supports the superiority of AIs over SERMs in this population, demonstrating not only reduced recurrence rates but also improved survival [21, 24]. Notably, this benefit extends to premenopausal women when AIs are combined with ovarian suppression, making them a standard option for high‐risk premenopausal cases [22]. However, AIs are associated with adverse effects such as bone loss and hot flashes, requiring long‐term monitoring of bone mineral density [25].

#### 2.1.3 SERDs

For patients who develop endocrine resistance, SERDs provide a critical therapeutic alternative by inducing ER degradation rather than mere receptor blockade [26, 27]. Fulvestrant—the first injectable SERD—has demonstrated efficacy in metastatic luminal BC, with a Phase III trial showing improved progression‐free survival (PFS) compared to exemestane in patients with *ESR1* mutations (a common driver of AI resistance) [26, 27].

Recent advances have focused on oral SERDs (e.g., elacestrant, Imlunestrant, giredestrant, AZD‐9833, and GDC‐9545) to address the inconvenience of injectable fulvestrant. In Phase II/III trials, oral SERDs achieved objective response rates (ORRs) of 13%–20% and median PFS of 4.5–7.8 months in patients refractory to prior endocrine therapy (with or without CDK4/6 inhibitors) [28]. As a result, elacestrant and imlunestrant showed meaningful activity in *ESR1*‐mutated metastatic BC, leading to their US Food and Drug Administration (FDA) approval as third‐line therapies [29, 30]. Moreover, recent data from the Phase III lidERA trial, presented at the 2025 San Antonio Breast Cancer Symposium (SABCS), demonstrated that giredestrant provided a statistically significant and clinically meaningful benefit in the adjuvant setting, reducing the risk of invasive disease by 30% compared with standard care (https://sabcs.org/events/general‐session‐1/).

A particularly innovative advancement is vepdegestrant (ARV‐471), an oral PROTAC ER degrader that harnesses the ubiquitin‒proteasome system to directly eliminate ER. In patients with ER‐positive, HER2‐negative advanced BC, vepdegestrant demonstrated significantly longer PFS than fulvestrant, but only in the subgroup with *ESR1* mutations, not in the overall population [31]. Given its potential, the FDA has granted the Fast Track designation for vepdegestrant as a monotherapy in ER‐positive/HER2‐negative locally advanced or metastatic BC previously treated with endocrine‐based regimens.

Ongoing clinical trials continue to explore these novel agents across various disease stages, potentially expanding the therapeutic landscape for luminal BC patients and addressing unmet needs in endocrine resistance.

### 2.2 Overcoming Resistance: CDK4/6 Inhibitors

Despite the efficacy of endocrine therapy, ∼30%–50% of luminal BC patients develop acquired resistance, often driven by dysregulation of the cell cycle. CDK4/6 play a central role in this process: estrogen signaling stimulates cyclin D1 expression, which, in turn, enhances CDK4/6 activity, resulting in hyperphosphorylation of retinoblastoma protein (RB1), thereby promoting G1‐to‐S phase transition and cell proliferation [32, 33]. This pathway remains active even after endocrine resistance develops, making CDK4/6 inhibition a pivotal strategy to restore treatment sensitivity.

#### 2.2.1 Clinical Efficacy of CDK4/6 Inhibitors

CDK4/6 inhibitors have become a cornerstone of combination therapy for HR‐positive/HER2‐negative BC, with multiple agents approved globally based on robust Phase III clinical data. These agents differ slightly in their clinical indications (e.g., metastatic vs. adjuvant settings, monotherapy vs. combination use) and efficacy profiles, which are critical for personalized treatment selection (Table 1).

**TABLE 1:** Main clinically approved cyclin‐dependent kinase 4/6 (CDK4/6) inhibitors for breast cancer treatment (as of 2025).

Agent	Clinical development (patient populations)	Clinical trials and efficacy	References
Palbociclib	HR‐positive and HER2‐negative locally advanced or metastatic breast cancer in combination with hormonal therapy	*PALOMA‐2*: Palbociclib + letrozole vs. placebo + letrozole (median PFS: 24.8 vs. 14.5 months)	[34]
		*PALOMA‐3*: Palbociclib + fulvestrant vs. placebo + fulvestrant (median PFS: 9.2 vs. 3.8 months)	[35]
Ribociclib	HR‐positive and HER2‐negative locally advanced or metastatic breast cancer in combination with hormonal therapy	*MONALEESA‐2*: Ribociclib + letrozole vs. placebo + letrozole (median PFS: 25.3 vs. 16.0 months)	[36]
		*MONALEESA‐3*:Ribociclib + fulvestrant vs. placebo + fulvestrant (median PFS: 20.5 vs. 12.8 months)	[37]
		*MONALEESA‐7*: Ribociclib + tamoxifen or NSAI + goserelin vs. placebo + tamoxifen or NSAI + goserelin (median PFS: 23.8 vs. 13.0 months)	[38]
	Adjuvant therapy for high‐risk, early‐stage HR‐positive, HER2‐negative breast cancer in combination with hormonal therapy	*NATALEE*: Ribociclib + NSAI vs. NSAI (3‐year iDFS: 90.4% vs. 87.1%)	[39]
Abemaciclib	Monotherapy for advanced HR‐positive and HER2‐negative breast cancer with disease progression following endocrine therapy and prior chemotherapy	*MONARCH 1*: Abemaciclib monotherapy: Objective response rate was 19.7% (95% CI: 13.3, 27.5) with a median response duration of 8.6 months (95% CI: 5.8, 10.2)	[40]
	HR‐positive and HER2‐negative advanced breast cancer in combination with hormonal therapy	*MONARCH 3*: Abemaciclib + anastrozole or letrozole vs. placebo + anastrozole or letrozole (median PFS: 28.2 vs. 14.8 months)	[41]
		*MONARCH 2*: Abemaciclib + fulvestrant vs. placebo + fulvestrant (median PFS: 16.4 vs. 9.3 months)	[42]
	Adjuvant therapy for high‐risk, early‐stage HR‐positive, HER2‐negative, and lymph node‐positive breast cancer in combination with hormonal therapy	*MONARCH E*: Abemaciclib + endocrine therapy vs. endocrine therapy (4‐year iDFS: 85.8% vs. 79.4%)	[43]
Dalpiciclib	Approved for HR‐positive and HER2‐negative breast cancer in combination with hormonal therapy	*DAWNA‐1*: Dalpiciclib + fulvestrant vs. placebo + fulvestrant (median PFS: 15.7 vs. 7.2 months)	[44]
		*DAWNA‐2*: Dalpiciclib + anastrozole or letrozole vs. placebo + dalpiciclib or letrozole (median PFS: 30.6 vs. 18.2 months)	[45]

##### 2.2.1.1 Palbociclib

The first evidence of CDK4/6 inhibitor efficacy came from the PALOMA‐1 trial (Phase II), where adding palbociclib to letrozole significantly improved PFS in ER‐positive/HER2‐negative advanced BC (20.2 vs. 10.2 months), leading to FDA approval in 2015 [46]. Subsequent Phase III trials (PALOMA‐2 and PALOMA‐3) confirmed its efficiency [34, 35]. However, adjuvant Phase III trials (PALLAS and PENELOPE‐B) failed to show significant invasive disease‐free survival (iDFS) benefits for palbociclib in early BC [47, 48].

##### 2.2.1.2 Ribociclib

Approved in 2017 based on the phase III MONALEESA‐2 trial, which showed a median PFS of 25.3 months with ribociclib + letrozole vs. 16.0 months with letrozole alone [36]. The Phase III MONALEESA‐3 trial confirmed its efficiency when combined with fulvestrant [37]. The Phase III MONALEESA‐7 trial showed its efficiency for premenopausal women with HR‐positive, advanced BC (ribociclib + tamoxifen or NSAI + goserelin vs. placebo + tamoxifen or NSAI + goserelin [median PFS: 23.8 vs. 13.0 months]) [38]. A subsequent Phase III adjuvant trial (NATALEE) demonstrated a 3.3% improvement in 3‐year iDFS (90.4% vs. 87.1%) in early high‐risk luminal BC, expanding its use to the adjuvant setting [39].

##### 2.2.1.3 Abemaciclib

Showed robust activity in the Phase II MONARCH 1 trial (single‐agent PFS of 6.0 months in refractory advanced BC) [40], Phase III MONARCH 3 confirmed its efficiency, where adding abemaciclib to letrozole significantly improved PFS in ER‐positive/HER2‐negative advanced BC (28.2 vs. 14.8 months) [41], and it was later approved in combination with fulvestrant for second‐line advanced therapy [42]. In the Phase III MonarchE trial, abemaciclib + endocrine therapy improved 5‐year iDFS by 7.6% (88.5% vs. 80.9%) and distant recurrence‐free survival (DRFS) by 6.7% in high‐risk node‐positive luminal BC, solidifying its role in early disease [43, 49]. Most recently, primary OS analysis from the MonarchE trial (with a median follow‐up of 6.3 years) yielded a statistically significant and clinically meaningful OS improvement of abemaciclib + endocrine therapy, and the abemaciclib + endocrine therapy regimen maintained a sustained benefit in both iDFS and DRFS at the 7‐year timepoint [50].

##### 2.2.1.4 Dalpiciclib

Approved by China's National Medical Products Administration (NMPA) in 2021 for advanced HR‐positive/HER2‐negative BC progressing after endocrine therapy based on a Phase III DAWNA‐1 trial, where dalpiciclib + fulvestrant significantly prolonged median PFS (15.7 vs. 7.2 months) compared to fulvestrant alone [44]. Furthermore, dalpiciclib in combination with letrozole or anastrozole may represent a novel first‐line standard of care for patients with HR‐positive, HER2‐negative advanced BC, offering a valuable alternative within the current treatment landscape [45].

### 2.3 Targeting Alternative Resistance Pathways: PI3K/Akt/mTOR Inhibitors

The PI3K/AKT/mTOR signaling pathway, a critical regulator of cell growth and survival, is dysregulated in over 70% of BCs, primarily through *PIK3CA* mutations, *AKT* mutations, or *PTEN* loss [51, 52]. Notably, these molecular alterations occur more frequently in HR‐positive BCs than in other subtypes, and the consequent hyperactivation of this pathway has been strongly associated with resistance to hormonal therapies [53]. Importantly, pharmacological inhibition of the PI3K/AKT/mTOR pathway has demonstrated the potential to overcome endocrine resistance [54], prompting the development of numerous targeted agents aimed at modulating this signaling cascade [55].

#### 2.3.1 mTOR Inhibitors

Everolimus, a first‐generation mTOR inhibitor, was the first agent in this class to be approved for luminal BC. The Phase III BOLERO‐2 trial showed that everolimus + exemestane significantly improved median PFS (7.8 vs. 3.2 months) in postmenopausal ER‐positive/HER2‐negative advanced BC patients who progressed on nonsteroidal AIs [56]. However, everolimus is associated with side effects such as stomatitis, fatigue, and hyperglycemia, limiting its long‐term use in some patients [57].

#### 2.3.2 PI3Kα Inhibitors

Given the high frequency of *PIK3CA* mutations in luminal BC, selective PI3Kα inhibitors have been developed to target this subtype of the PI3K enzyme.

##### 2.3.2.1 Alpelisib

Approved by the FDA in 2019 based on the Phase III SOLAR‐1 trial, alpelisib + fulvestrant improved median PFS (11.0 vs. 5.7 months) in *PIK3CA*‐mutated ER‐positive/HER2‐negative metastatic BC [58]. Although the prespecified statistical significance boundary for OS was not crossed, a numerical improvement of 7.9 months in median OS was observed with the addition of alpelisib to fulvestrant in patients with *PIK3CA*‐mutated, HR‐positive/HER2‐negative advanced BC [59]. This treatment benefit was confined to *PIK3CA*‐mutated cases, underscoring the critical role of biomarker testing.

##### 2.3.2.2 Inavolisib

The FDA granted approval on October 10, 2024, for inavolisib in combination with palbociclib and fulvestrant for patients with endocrine‐resistant, *PIK3CA*‐mutated, ER‐positive/HER2‐negative locally advanced or metastatic BC. This decision was supported by the Phase III INAVO120 trial, where triple therapy significantly improved median PFS to 16.5 months, compared to 9.3 months with palbociclib–fulvestrant alone, highlighting its efficacy within a multi‐agent regimen [60, 61]. Moreover, the inavolisib‐based combination also demonstrated a significant OS benefit, with a median OS of 34 versus 27 months in the placebo plus palbociclib–fulvestrant group [60].

#### 2.3.3 AKT Inhibitors

The therapeutic arsenal has further expanded with the development of AKT inhibitors, including capivasertib and ipatasertib, which have demonstrated promising antitumor activity in both preclinical and clinical studies [55, 62, 63]. Notably, capivasertib, a selective AKT inhibitor, was approved by the FDA in 2023 based on the Phase III CAPItello‐291 trial: in patients with ER‐positive/HER2‐negative advanced BC harboring PIK3CA/AKT1/PTEN alterations, capivasertib + fulvestrant improved median PFS (7.2 vs. 3.6 months) and OS (20.5 vs. 14.3 months) compared to fulvestrant alone [64]. Another AKT inhibitor, ipatasertib, showed promising activity in Phase II trials (e.g., combination with paclitaxel in *PIK3CA*‐mutated BC), although it has not yet received regulatory approval [63]. These sequential advancements underscore the critical importance of the PI3K/AKT/mTOR pathway in HR‐positive BC and highlight the ongoing evolution of targeted therapies to address endocrine resistance.

### 2.4 Emerging Targets and Strategies

#### 2.4.1 HDAC Inhibitors

HDACs serve as critical epigenetic regulators by removing acetyl groups from histones, resulting in chromatin condensation and transcriptional repression [65]. In luminal BC, HDACs interact directly with ER‐α to suppress ER transcriptional activity, promoting endocrine resistance [66, 67]. HDAC inhibitors (HDACis), such as entinostat and vorinostat, reverse this effect by restoring ER expression and sensitivity to endocrine therapy.

Entinostat (a Class I HDACi) has shown promise in combination with exemestane. The Phase II ENCORE301 trial reported improved median PFS (4.3 vs. 2.3 months) and OS (28.1 vs. 19.8 months) with entinostat + exemestane in patients who progressed on nonsteroidal AIs but were naïve to CDK4/6 inhibitors [68]. The Phase III E2112 trial (including patients pretreated with CDK4/6 inhibitors or fulvestrant) failed to replicate OS benefits [69]. The Chinese Phase III EOC103A3101 trial confirmed a median PFS improvement (5.8 vs. 3.6 months) with entinostat + exemestane in a Chinese patient population [70]. This led to NMPA approval of entinostat in 2024 for endocrine‐resistant HR‐positive advanced BC, making it a valuable option in Asian patients.

Another HDACi, vorinostat, showed activity in combination with tamoxifen in Phase II trials (ORR = 18%), although it has not advanced to Phase III due to limited efficacy compared to newer agents [71].

#### 2.4.2 Steroid Sulfatase Inhibitors

Steroid sulfatase (STS) is a key enzyme in steroid metabolism responsible for converting inactive steroid sulfates, specifically estrone sulfate (E1S), to estrone (E1) and dehydroepiandrosterone sulfate (DHEAS) to dehydroepiandrosterone (DHEA), into their active forms, which can then be further metabolized into estrogens. Due to its role in local estrogen production, STS is closely linked to tumor growth in hormone‐responsive BC [72]. Notably, STS expression and activity are significantly elevated in ERα‐positive BC, making it a potential target to reduce estrogen bioavailability [73].

Irosustat (the first‐generation STS inhibitor) showed good tolerability in phase II trials: when combined with AIs, it improved median PFS (6.4 vs. 4.8 months) in postmenopausal ER‐positive locally advanced or metastatic BC [74]. However, despite these promising early results, irosustat ultimately failed to advance into Phase III clinical trials, likely due to insufficient efficacy as a monotherapy and the shifting treatment landscape favoring more potent targeted therapies such as CDK4/6 inhibitors and next‐generation endocrine agents. Further research may explore STS inhibition in combination strategies or specific patient subsets where its mechanism could offer a therapeutic advantage.

#### 2.4.3 AR

AR modulation shows therapeutic potential in HR‐positive disease. In a Phase II trial, the oral selective AR modulator enobosarm demonstrated antitumor activity in patients with AR‐positive/ER‐positive/HER2‐negative advanced BC, indicating the therapeutic potential of AR activation in this population [75]. This finding led to the FDA granting fast‐track designation to enobosarm for AR‐positive/ER‐positive/HER2‐negative metastatic BC. Furthermore, research indicates that combining AR inhibition with endocrine therapy may enhance treatment efficacy in HR‐positive disease. Although the addition of enzalutamide to exemestane did not improve PFS in an unselected population, subgroup analysis revealed a potential benefit in endocrine therapy‐naïve patients with high *AR* mRNA expression, particularly when combined with low *ESR1* mRNA levels [76]. This highlights the importance of biomarker‐driven patient selection for AR‐targeted therapies across BC subtypes.

### 2.5 Future Directions

Emerging strategies in luminal BC include (1) combining PROTAC ER degraders (e.g., vepdegestrant) with CDK4/6 inhibitors to target *ESR1*‐mutated and RB1‐intact tumors; (2) using dual PI3K/mTOR inhibitors (e.g., gedatolisib) to overcome resistance to single‐agent PI3K inhibitors; (3) employing ADCs such as datopotamab deruxtecan (Dato‐DXd), which is FDA‐approved for unresectable or metastatic HR‐positive/HER2‐negative BC after prior endocrine therapy and chemotherapy in the metastatic setting [77]; and (4) employing liquid biopsy‐guided dynamic treatment adjustment (e.g., monitoring *ESR1* mutations in circulating tumor DNA [ctDNA] to switch therapies early) [78]. These approaches aim to further personalize treatment and extend durable responses in luminal BC.

## 3 Targeting Therapies for HER2‐Positive BC

**FIGURE 4:**
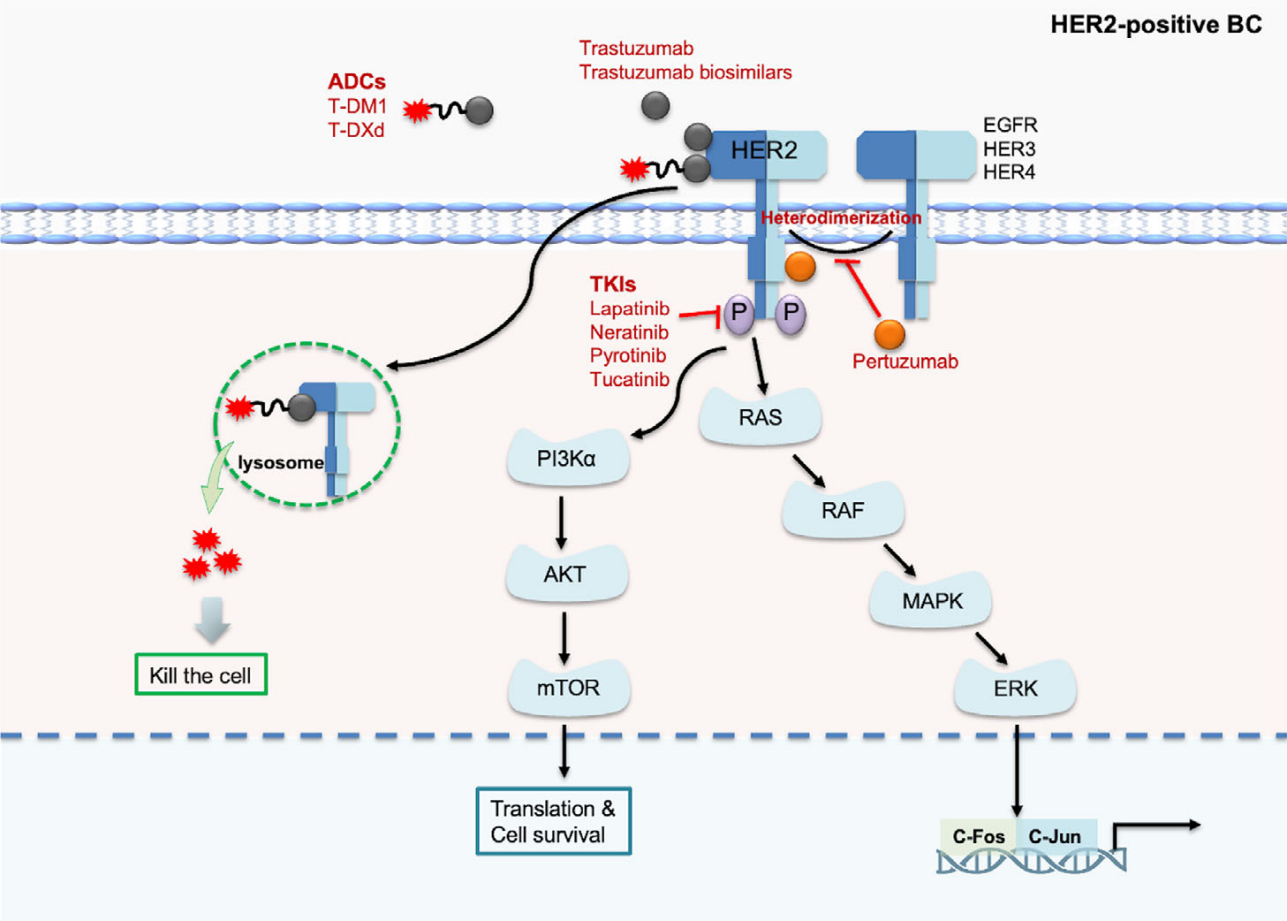
Evolution and mechanisms of anti‐HER2 targeted therapy for HER2‐positive breast cancer Depicts the action mechanisms of anti‐HER2 agents, including monoclonal antibodies (mAbs), antibody‒drug conjugates (ADCs), and tyrosine kinase inhibitors (TKIs), in the treatment of HER2‐positive breast cancer.

Historically, HER2‐positive BC was considered one of the most aggressive subtypes due to the association between HER2 amplification/overexpression and malignant tumor behavior, as well as significantly poor survival outcomes [79]. However, the therapeutic landscape for this disease was revolutionized with the approval of trastuzumab, the first HER2‐targeted mAb, which transformed HER2‐positive BC into a subtype with markedly improved prognosis. Since then, rapid advancements in HER2‐targeted therapies, including pertuzumab, tucatinib, and T‐DXd, have further enhanced survival benefits for patients [6] (Table 2). The remarkable clinical efficacy demonstrated across multiple HER2‐targeted trials has also spurred extensive research efforts to develop novel and more potent targeted therapies (Figure 4).

**TABLE 2:** Main clinically approved anti‐human epidermal growth factor receptor 2 (HER2) agents for breast cancer treatment (as of 2025).

Agent	Clinical development (patient populations)	Clinical trials and efficacy	References
Trastuzumab (HER2 mAbs)	Neoadjuvant therapy for HER2‐positive breast cancer in combination with chemotherapy	*NOAH*: Trastuzumab + chemotherapy vs. chemotherapy (tpCR: 38% vs.19%; 3‐year EFS: 71% vs. 56%)	[80]
	Adjuvant therapy for HER2‐positive breast cancer in combination with chemotherapy	*HERA*: Trastuzumab after adjuvant chemotherapy vs. adjuvant chemotherapy (10‐year DFS: 69% vs.63%; 10‐year OS: 79% vs. 73%)	[81]
		*NSABP B‐31*/*NCCTG N9831*: Trastuzumab + adjuvant chemotherapy vs. adjuvant chemotherapy (10‐year DFS: 73.7% vs. 62.2%; 10‐year OS: 84% vs. 75.2%)	[82]
	HER2‐positive metastatic breast cancer	*H0648g*: Trastuzumab + chemotherapy vs. chemotherapy (DFS: 7.4 vs. 4.6 months; OS: 25.1 vs. 20.3 months)	[83]
Pertuzumab (HER2 mAbs)	Neoadjuvant therapy for HER2‐positive breast cancer in combination with trastuzumab and chemotherapy	*Neosphere*: Trastuzumab + docetaxel vs. pertuzumab + trastuzumab + docetaxel vs. pertuzumab + trastuzumab vs. pertuzumab + docetaxel (pCR: 29.0% vs. 45.8% vs. 16.8% vs. 24.0%)	[84]
	Adjuvant therapy for HER2‐positive breast cancer in combination with trastuzumab and chemotherapy	*APHINITY*: Pertuzumab + trastuzumab + chemotherapy vs. trastuzumab + chemotherapy (6‐year iDFS: 91% vs. 88%)	[85]
Margetuximab (HER2 mAbs)	For HER2‐positive metastatic breast cancer in combination with trastuzumab and chemotherapy	*CLEOPATRA*: Pertuzumab + trastuzumab + chemotherapy vs. trastuzumab + chemotherapy (OS: 57.1 vs. 40.8 months)	[86]
Trastuzumab emtansine (T‐DM1) (HER2 ADC)	For HER2‐positive breast cancer who have received two or more prior anti‐HER2 regimens in in combination with chemotherapy	*SOPHIA*: Margetuximab + chemotherapy vs. trastuzumab + chemotherapy (PFS: 5.8 vs. 4.9 months; OS: 21.6 vs. 21.9 months)	[87]
	For HER2‐positive early breast cancer patients who have residual invasive disease after neoadjuvant treatment with a taxane and trastuzumab	*KATHERINE*: T‐DM1 vs. trastuzumab (3‐year iDFS: 88.3% vs. 77.0%)	[88]
	For metastatic HER2‐positive breast cancer	*EMILIA*: T‐DM1 vs. lapatinib + capecitabine (PFS: 9.6 vs. 6.4 months; OS: 30.9 vs. 25.1 months)	[89]
Trastuzumab deruxtecan (T‐DXd, DS‐8201a) (HER2 ADC)	For HER2‐positive advanced breast cancer patients who have received two or more anti‐HER2‐based regimens	*DESTINY‐Breast01* (phase II): PFS: 16.4 months	[90]
	For HER2‐positive metastatic breast cancer patients who were refractory or resistant to trastuzumab emtansine	*DESTINY‐Breast02*: T‐DXd vs. physician's choice (PFS: 17.8 vs. 6.9 months)	[91]
		*DESTINY‐Breast03*: T‐DXd vs. T‐DM1 (PFS: 28.8 vs. 6. months)	[92]
	For HER2‐low/ultralow advanced breast cancer	*DESTINY‐Breast04*: T‐DXd vs. physician's choice (PFS: 10.1 vs. 5.4 months; OS: 23.9 vs. 17.5 months)	[93]
		*DESTINY‐Breast06*: T‐DXd vs. physician's choice (PFS: 13.2 vs. 8.1 months)	[94]
	For HER2‐positive early breast cancer patients who have residual invasive disease after neoadjuvant treatment	*DESTINY‐Breast05*: T‐DXd vs. T‐DM1 (3‐year iDFS: 88.3% vs. 77.0%)	[95]
	For HER2‐positive advanced or metastatic breast cancer with no previous therapy	*DESTINY‐Breast09*: T‐DXd + pertuzumab vs. taxane + trastuzumab + pertuzumab (PFS: 40.7 vs. 26.9 months)	[96]
	Neoadjuvant therapy for HER2‐positive breast cancer	*DESTINY‐Breast11*: T‐DXd‐THP vs. ddAC‐THP (tpCR: 67.3% vs.56.3%; 2‐year EFS: 96.9% vs. 93.1%)	[97]
Lapatinib (TKI)	For HER‐2‐positive metastatic breast cancer in combination with capecitabine	*EGF100151*: Lapatinib + capecitabine vs. capecitabine (PFS: 8.4 vs. 4.4 months)	[98]
Neratinib (TKI)	Adjuvant treatment for early‐stage HER2‐opositive breast cancer, to follow adjuvant trastuzumab‐based therapy	*ExteNET*: Extended adjuvant therapy with neratinib for a year vs. placebo (5‐year iDFS: 90.2% vs. 87.7%)	[99]
	For HER2‐positive metastatic breast cancer in combination with capecitabine	*NALA*: Lapatinib + capecitabine vs. neratinib + capecitabine (PFS: 8.8 vs. 6.6 months)	[100]
Pyrotinib (TKI)	For HER2‐positive metastatic breast cancer in combination with capecitabine	*PHOEBE*: Pyrotinib + capecitabine vs. lapatinib + capecitabine (PFS: 12.5 vs. 6.8 months)	[101]
	Adjuvant therapy for HER2‐positive breast cancer in combination with trastuzumab and chemotherapy	*PHEDRA*: Pyrotinib + trastuzumab + docetaxel vs. placebo + trastuzumab + docetaxel (tpCR: 41% vs. 22%; 3‐year EFS: 71% vs. 56%)	[102]
Tucatinib (TKI)	For HER2‐positive metastatic breast cancer in combination with capecitabine	*HER2CLIMB*: Tucatinib + trastuzumab + capecitabine vs. placebo + trastuzumab + capecitabine (CNS‐PFS: 13.9 vs 5.6 months)	[103]

### 3.1 The Evolution of Anti‐HER2 Therapy: From mAbs to ADCs

Anti‐HER2 therapy has undergone three key generations, each addressing limitations of the prior and improving clinical outcomes by enhancing target specificity, cytotoxic potency, and coverage of resistant or heterogeneous tumors.

#### 3.1.1 First Generation: mAbs—Foundational Targeting of the HER2 Extracellular Domain

##### 3.1.1.1 Trastuzumab

The first breakthrough in HER2‐positive BC treatment came with trastuzumab, the first humanized anti‐HER2 mAb. Mechanistically, it binds to subdomain IV of the HER2 extracellular domain (ECD), suppressing intracellular signaling pathways, inhibiting tumor cell proliferation, and mediating antibody‐dependent cellular cytotoxicity (ADCC) [104]. Pivotal clinical trials established trastuzumab as the cornerstone of HER2‐positive BC therapy:

*Neoadjuvant setting*: The Phase III NOAH trial demonstrated that in patients with HER2‐positive locally advanced or inflammatory BC, the addition of 1 year of trastuzumab (initiated as neoadjuvant and continued as adjuvant therapy) to neoadjuvant chemotherapy was associated with nearly doubled rates of pathological complete response (43% vs. 22%) and significantly improved 3‐year event‐free survival (71% vs. 56%) compared with chemotherapy alone [80].

*Adjuvant setting*: The Phase III HERA trial showed that trastuzumab added after adjuvant chemotherapy reduced the risk of recurrence by 46% and improved 10‐year OS to 79% in early‐stage HER2‐positive BC [81]. The phase III NSABP B‐31 and NCCTG N9831 trials further confirmed that trastuzumab + taxane‐based chemotherapy improved 10‐year DFS by 11.5% and 10‐year OS by 8.8% compared to chemotherapy alone [82].

*Metastatic setting*: Data from the pivotal H0648g Phase III trial in the metastatic setting indicated that the addition of trastuzumab significantly prolonged PFS (median: 7.4 vs. 4.6 months) and improved OS (median: 25.1 vs. 20.3 months) [83].

##### 3.1.1.2 Pertuzumab

Approximately 30% of patients develop acquired resistance to trastuzumab, driven by mechanisms such as cleavage of the HER2 ECD (generating a constitutively active intracellular fragment), upregulation of alternative receptors (e.g., HER3), or hyperactivation of downstream pathways (e.g., PI3K/AKT) [105], highlighting the need for additional antibodies targeting distinct HER2 epitopes to achieve synergistic antitumor effects [106]. This led to the development of pertuzumab, a second‐generation anti‐HER2 mAb that binds subdomain II of HER2, preventing heterodimerization with HER1 (EGFR), HER3, and HER4, thereby blocking downstream oncogenic signaling [107]. Clinical trials validated its synergistic effect with trastuzumab:

*Neoadjuvant setting*: The Phase II NeoSphere trial demonstrated that in patients with locally advanced, inflammatory, or early‐stage HER2‐positive BC, four cycles of neoadjuvant pertuzumab, trastuzumab, and docetaxel significantly improved the pCR rate in the breast by 16.8% compared to trastuzumab plus docetaxel alone [84].

*Adjuvant setting*: In the Phase III APHINITY trial, the addition of pertuzumab to trastuzumab and chemotherapy was associated with a 5% reduction in iDFS events in high‐risk early HER2‐positive BC, with the benefit driven primarily by patients with node‐positive disease [85].

*Metastatic setting*: The Phase III CLEOPATRA trial showed that pertuzumab + trastuzumab + docetaxel improved the median OS to 57.1 months (vs. 40.8 months with trastuzumab + docetaxel), with a 34% reduction in the risk of death [86].

To improve patient convenience, subcutaneous formulations of trastuzumab (e.g., Herzuma biosimilar) and fixed‐dose combinations (e.g., subcutaneous pertuzumab‐trastuzumab) have been developed. These formulations show equivalent pharmacokinetics, efficacy, and safety to intravenous administration while reducing treatment time (from ∼90 to ∼5 min) and healthcare burden [18, 108, 109].

##### 3.1.1.3 Margetuximab

Margetuximab‐cmkb is an fragment crystallizable (Fc)‐engineered anti‐HER2 mAb that binds to the same epitope as trastuzumab while demonstrating comparable antiproliferative effects [110]. The drug received FDA approval based on the primary analysis of the Phase III SOPHIA trial (NCT02492711), which showed improved PFS by central review in patients with HER2‐positive metastatic BC who had received ≥ 2 prior anti‐HER2 regimens (including at least one for metastatic disease) [87]. However, the final OS analysis with updated safety data revealed comparable safety profiles between margetuximab and trastuzumab but failed to demonstrate a statistically significant OS advantage for margetuximab [111].

##### 3.1.1.4 Trastuzumab Biosimilars

Trastuzumab biosimilars represent a class of biologically derived agents manufactured through living cell systems that demonstrate comparable pharmacokinetic and pharmacodynamic profiles to the reference HER2‐targeted mAb. These biosimilars are required to demonstrate no clinically meaningful differences from the originator product in terms of safety, purity, or therapeutic potency, although they may contain minor variations in clinically inactive components as permitted by regulatory standards (FDA guidance 2018) [112]. Currently, six trastuzumab biosimilars have received FDA approval for use in the U.S. market: trastuzumab‐dkst, trastuzumab‐pkrb, trastuzumab‐dttb, trastuzumab‐qyyp, trastuzumab‐anns, and trastuzumab‐strf [113, 114]. Despite receiving endorsement in clinical guidelines and demonstrating equivalent efficacy and safety profiles, the adoption of trastuzumab biosimilars in clinical practice has progressed slowly. This delayed uptake has been primarily attributed to several key factors: limited physician familiarity with biosimilar products, persistent perceptions regarding potential differences in clinical performance, variable coverage policies among commercial payers, and established preferences for the reference product among healthcare providers [115]. These implementation challenges have persisted despite the potential for biosimilars to enhance treatment accessibility and reduce healthcare costs through increased competition in the biologics market.

#### 3.1.2 Generation: ADCs—Precision Delivery of Cytotoxic Payloads

To overcome mAb resistance and enhance potency, ADCs were developed as a “guided missile” system: a tumor‐targeting mAb (trastuzumab) is conjugated to a highly potent cytotoxic payload via a stable linker, enabling selective delivery of toxins to HER2‐positive cells while sparing healthy tissue [116].

##### 3.1.2.1 Trastuzumab Emtansine (T‐DM1)

T‐DM1 stands as the first FDA‐approved anti‐HER2 ADC, comprising trastuzumab conjugated to the maytansinoid derivative DM1 through a stable thioether linker, with an average drug‐to‐antibody ratio (DAR) of 3.5. Preclinical studies demonstrated T‐DM1's ability to induce cytotoxicity in HER2‐positive cell lines irrespective of their sensitivity to trastuzumab or lapatinib, highlighting its distinct mechanism of action [117, 118]. Clinically, T‐DM1 addressed critical unmet needs:

*Adjuvant setting*: The Phase III KATHERINE trial showed that T‐DM1 reduced the risk of recurrence by 50% in early HER2‐positive BC patients with residual invasive disease after neoadjuvant therapy (a population at high risk of relapse), with a 3‐year iDFS of 88.3% (vs. 77.0% with trastuzumab) [88, 119].

*Metastatic setting*: The Phase III EMILIA trial demonstrated that T‐DM1 improved median PFS to 9.6 months (vs. 6.4 months with lapatinib + capecitabine) and OS to 30.9 months (vs. 25.1 months) in trastuzumab‐pretreated metastatic HER2‐positive BC [89]. A post hoc analysis of the KAMILLA trial (a Phase IIIb study of T‐DM1 in metastatic HER2‐positive BC) showed that T‐DM1 achieved a 28% ORR in patients with active brain metastases, addressing a major clinical challenge of HER2‐positive BC [120].

#### 3.1.3 Third Generation: Next‐Generation ADCs—Enhancing Potency and Overcoming Heterogeneity

##### 3.1.3.1 Trastuzumab Deruxtecan (T‐DXd, DS‐8201a)

The limitations of T‐DM1 (e.g., low DAR, non‐cleavable linker restricting payload release) led to the development of T‐DXd, a next‐generation ADC that revolutionized HER2‐positive BC treatment. T‐DXd consists of trastuzumab conjugated to deruxtecan (a topoisomerase I inhibitor) via a cleavable tetrapeptide linker, with an exceptionally high DAR of 8—nearly 2.5 times that of T‐DM1 [121]. This design enhances payload delivery to tumors, and the cleavable linker enables “bystander killing” of nearby HER2‐low or HER2‐negative cells (via payload diffusion), addressing intratumoral heterogeneity. Key clinical trials established T‐DXd as a transformative agent:

*Refractory metastatic HER2‐positive BC*: The Phase II DESTINY‐Breast01 trial led to the first approval of T‐DXd in 2019 for patients with HER2‐positive advanced BC who had received two or more anti‐HER2‐based regimens [90]. The Phase III DESTINY‐Breast 02 trial showed the favorable benefit‐risk profile of T‐DXd in patients with HER2‐positive metastatic BC who had progressed on prior therapies. As assessed by blinded independent central review, the median PFS was 17.8 months in the T‐DXd group, compared to 6.9 months in the treatment of physician's choice group [91]. The Phase III DESTINY‐Breast03 trial demonstrated a further remarkable advancement, where T‐DXd outperformed T‐DM1. It extended the median PFS to 28.8 months from 6.8 months and achieved an ORR of 79.7% versus 34.2% with T‐DM1, representing an unprecedented clinical benefit in this heavily pretreated population [92]. DESTINY‐Breast12 evaluated T‐DXd in advanced HER2‐positive BC with or without brain metastases. In patients with brain metastases, the 12‐month PFS rate was 61.6%, and the 12‐month central nervous system (CNS) PFS rate was 58.9%, confirming substantial intracranial activity [122]. Most notably, the recently published DESTINY‐Breast09 trial assessed T‐DXd in patients with previously untreated HER2‐positive advanced or metastatic BC. When used as first‐line therapy, T‐DXd combined with pertuzumab significantly reduced the risk of progression or death compared to THP (median PFS: 40.7 vs. 26.9 months), with no new safety signals observed [96].

*HER2‐low/ultralow BC*: The DESTINY‐Breast04 trial fundamentally expanded the therapeutic landscape for metastatic BC by establishing T‐DXd as an effective treatment for HER2‐low disease (IHC 1+ or IHC 2+/ISH−: Immunohistochemical staining for HER2 showing weak [1+] or moderate [2+] intensity, with in situ hybridization [ISH] confirming no HER2 gene amplification), a subtype previously excluded from anti‐HER2 therapy. The trial demonstrated that T‐DXd significantly improved median PFS to 10.1 months compared to 5.4 months with physician's choice chemotherapy and OS to 23.9 versus 17.5 months [108]. This finding represented a paradigm shift, redefining the boundaries of targetable HER2 expression and benefiting approximately 50% of all patients previously categorized as HER2‐negative [93]. Furthermore, the DESTINY‐Breast06 trial investigated T‐DXd in patients with HR‐positive, HER2‐low, or HER2‐ultralow (defined as IHC 0 with membrane staining) metastatic BC who had received one or more lines of endocrine‐based therapy and were chemotherapy‐naïve in the metastatic setting. In the primary analysis, T‐DXd provided a statistically significant improvement in PFS compared to physician's choice chemotherapy in the HER2‐low population, with a hazard ratio of 0.62 and median PFS of 13.2 versus 8.1 months [94].

*HER2‐positive early BC*: The Phase III DESTINY‐Breast05 trial is comparing T‐DXd with T‐DM1 as adjuvant therapy for patients who did not achieve a pCR after neoadjuvant treatment. Recently, presented data show a 3‐year DFS rate of 92.3% with T‐DXd versus 83.5% with T‐DM1, corresponding to an absolute improvement of 8.8% [95].

*Ongoing trials in neoadjuvant settings*: In the neoadjuvant setting, the Phase III DESTINY‐Breast11 trial is investigating whether T‐DXd alone or T‐DXd followed by THP can replace the standard TCbHP regimen. The results presented at ESMO 2025 indicated that the T‐DXd–THP regimen led to a statistically significant and clinically meaningful increase in pCR rates compared with ddAC‐THP (tpCR: 67.3% vs. 56.3%; 2‐year EFS: 96.9% vs. 93.1%), along with an improved safety profile [97].

### 3.2 Small Molecule Inhibitors: TKIs

While mAbs and ADCs target the HER2 ECD, TKIs act on the intracellular catalytic kinase domain of HER2, blocking receptor phosphorylation and downstream signaling [123]. Their small molecular size enables penetration of the blood‒brain barrier (BBB)—a critical advantage for treating HER2‐positive BC brain metastases—and activity against trastuzumab‐resistant tumors with intracellular HER2 alterations (e.g., *HER2* mutations). Four TKIs have emerged as key agents in HER2‐positive BC therapy.

#### 3.2.1 Lapatinib—Dual HER1/HER2 Inhibition for Trastuzumab Resistance and Brain Metastases

Lapatinib is an orally administered 4‐anilinoquinazoline derivative that demonstrates dual inhibitory activity against both HER1 (EGFR) and HER2 tyrosine kinases through competitive binding to the ATP‐binding site of the intracellular domain. Notably, lapatinib maintains its therapeutic efficacy even in trastuzumab‐resistant HER2‐positive tumors, as demonstrated by both in vitro and in vivo studies [124, 125, 126]. Clinical trials investigating lapatinib in HER2‐positive early breast cancer (EBC) have yielded promising results, with comprehensive data summarized in recent reviews [127]. The phase clinical trial (EGF100151) led to the FDA approval of lapatinib in combination with capecitabine for HER‐2‐positive metastatic BC that progressed after trastuzumab‐based therapy. This study showed that the lapatinib‐based regimen significantly improved median PFS (8.4 vs. 4.4 months) [98]. A particularly significant therapeutic advantage of lapatinib lies in its activity against CNS metastases, which represent a major clinical challenge in HER2‐positive BC management [128]. The LANDSCAPE trial (a single‐group Phase II study) evaluated lapatinib + capecitabine in patients with untreated brain metastases from HER2‐positive metastatic BC, achieving a 65.9% intracranial ORR and median intracranial PFS of 15.7 months [129]. A multicenter Phase II trial further confirmed lapatinib's activity in pretreated brain metastases, with a 20.5% intracranial ORR [130].

#### 3.2.2 Neratinib—Irreversible Pan‐HER Inhibition for Extended Adjuvant and Metastatic Settings

Neratinib (HKI‐272) represents a clinically significant irreversible TKI that broadly targets the HER receptor family, including EGFR (HER1), HER2, and HER4 [131]. This pan‐HER inhibition profile confers several unique therapeutic advantages. First, neratinib demonstrates robust antitumor activity even in trastuzumab‐resistant cell lines while also exhibiting synergistic effects when combined with trastuzumab [132]. Clinically, the ExteNET trial (a Phase III randomized trial) evaluated neratinib versus placebo after 1 year of trastuzumab‐based adjuvant therapy in early HER2‐positive BC. Neratinib improved 5‐year invasive DFS by 2.5% (90.2% vs. 87.7%), with a more pronounced benefit in HR‐positive patients (5‐year iDFS: 91.2% vs. 86.8%) [99, 133]. In advanced HER2‐positive metastatic BC, the Phase III NALA trial led to the FDA approval of neratinib in combination with capecitabine for patients previously treated with two or more anti‐HER2 regimens. This study showed that the neratinib‐based regimen significantly improved median PFS (8.8 vs. 6.6 months) and reduced the risk of CNS progression by 40% compared to lapatinib plus capecitabine [100]. Notably, neratinib possesses a distinctive pharmacological characteristic: it remains active against tumor cell lines harboring somatic *HER2* mutations (e.g., HER2 L869R, G776V), regardless of HER2 amplification status. This unique property suggests that neratinib may overcome resistance mechanisms that limit the effectiveness of other HER2‐targeted therapies, potentially expanding its clinical utility beyond conventional HER2‐positive disease [132].

#### 3.2.3 Pyrotinib—Pan‐HER TKI Approved for Chinese Patients With Trastuzumab Resistance

Pyrotinib is another oral, irreversible pan‐HER TKI that has gained approval from NMPA for the treatment of HER2‐positive metastatic BC in patients who have previously received trastuzumab and chemotherapy. This approval was based on compelling clinical evidence demonstrating that pyrotinib combined with capecitabine significantly prolonged PFS compared to the lapatinib‐capecitabine regimen while maintaining an acceptable safety profile [101]. The therapeutic potential of pyrotinib extends across multiple treatment settings. In the neoadjuvant setting, the Phase III PHEDRA trial revealed significant clinical benefits when pyrotinib was combined with trastuzumab and docetaxel for patients with HER2‐positive early or locally advanced BC [134]. Furthermore, emerging data suggest that extended adjuvant pyrotinib therapy following standard trastuzumab‐based treatment may offer particular advantages for high‐risk HER2‐positive BC patients. Ongoing follow‐up studies are currently evaluating the long‐term survival benefits of this treatment approach [102]. These clinical findings position pyrotinib as a valuable addition to the HER2‐targeted therapy arsenal, with demonstrated efficacy in metastatic, neoadjuvant, and adjuvant settings. Its unique irreversible binding mechanism and pan‐HER inhibitory activity may provide distinct advantages in overcoming resistance to existing therapies.

#### 3.2.4 Tucatinib—Highly Selective HER2 Inhibition for Brain Metastases

Tucatinib is a highly selective, orally administered TKI that demonstrates remarkable specificity for HER2, showing > 1000‐fold greater potency against HER2 compared to EGFR [135, 136]. Preclinical studies have established its superior CNS penetration capability relative to lapatinib, making it particularly effective against brain metastases, a common and challenging complication of HER2‐positive MBC. These promising preclinical findings were clinically validated in the pivotal HER2CLIMB trial, which demonstrated significant survival benefits with the tucatinib–trastuzumab–capecitabine combination in patients with heavily pretreated HER2‐positive MBC [137, 138]. Importantly, the regimen showed clinically meaningful efficacy in patients with brain metastases, including those with active CNS disease. These compelling results led to FDA's 2020 approval of tucatinib for the treatment of HER2‐positive MBC, specifically including patients with CNS metastases, thereby addressing a critical unmet need in this patient population. The combination's proven activity against both systemic and CNS metastasis solidifies tucatinib as a valuable therapeutic option for advanced HER2‐positive BC [139].

### 3.3 Tackling Heterogeneity and Resistance: Novel ADCs and Combination Strategies

HER2‐positive BC exhibits significant intratumoral heterogeneity (e.g., coexistence of HER2‐high and HER2‐low cells) and acquired resistance to standard anti‐HER2 therapies—two major barriers to long‐term remission. Recent advances focus on novel ADCs (to address heterogeneity) and rational combinations (to target resistance mechanisms).

#### 3.3.1 Novel ADCs for Heterogeneous and Resistant HER2‐Positive BC

Next‐generation ADCs are designed to overcome the limitations of T‐DM1 and T‐DXd, with innovations in target selection, linker stability, and payload potency:

##### 3.3.1.1 HER3‐Targeted ADCs

HER3 is the preferred dimerization partner for HER2, and its upregulation is a major mechanism of trastuzumab and T‐DM1 resistance [140]. Patrituximab deruxtecan (HER3‐DXd) is an ADC consisting of a HER3‐targeting mAb conjugated to deruxtecan (same payload as T‐DXd). A Phase I trial showed that it achieved a 32% ORR in HER2‐positive BC patients pretreated with T‐DXd, supporting its role in T‐DXd‐resistant disease [140].

##### 3.3.1.2 Dual‐Targeted ADCs

SYD985 (trastuzumab duocarmazine) is an ADC that binds HER2 and delivers a DNA‐alkylating payload (duocarmycin) via a cleavable linker. A Phase II trial in T‐DM1‐resistant HER2‐positive BC showed a 31% ORR and median PFS of 5.1 months, with activity in HER2‐low subpopulations [141]. These findings were confirmed in the Phase III TULIP trial, where SYD985 significantly improved median PFS compared to physician's choice of treatment (7.0 vs. 4.9 months). The PFS benefit was consistent across most predefined subgroups. At the first interim analysis, a trend toward improved OS was observed with SYD985 (median OS: 20.4 vs. 16.3 months), although not statistically significant [141].

#### 3.3.2 Combination Strategies to Overcome Resistance

Resistance to anti‐HER2 therapy often arises from activation of alternative signaling pathways (e.g., PI3K/AKT/mTOR) or bypass via other HER family members. Rational combinations target these pathways synergistically:

##### 3.3.2.1 Dual HER2 Blockade + Chemotherapy

The standard of care for early and metastatic HER2‐positive BC remains dual HER2 blockade (trastuzumab + pertuzumab) + chemotherapy, as validated by the CLEOPATRA (metastatic) and APHINITY (adjuvant) trials [85, 86]. This combination addresses resistance via complementary mechanisms (trastuzumab blocks HER2 signaling, pertuzumab prevents dimerization) and is now the backbone of curative‐intent therapy for early HER2‐positive BC.

##### 3.3.2.2 Anti‐HER2 Agents + PI3K/AKT/mTOR Inhibitors

The hyperactivation of the PI3K/AKT/mTOR pathway has been identified as a key mechanism underlying acquired resistance to trastuzumab in HER2‐positive BC [142]. This understanding has prompted extensive investigation into combination strategies pairing PI3K/AKT/mTOR pathway inhibitors with trastuzumab to overcome therapeutic resistance. Clinical studies have demonstrated promising results with PI3K inhibitors, where buparlisib and pilaralisib showed feasible safety profiles and encouraging efficacy when combined with lapatinib, trastuzumab alone, or trastuzumab plus paclitaxel in pretreated HER2‐positive advanced BC patients [143, 144, 145]. Similarly, the AKT inhibitor MK‐2206 exhibited favorable antitumor activity in combination with either trastuzumab monotherapy or trastuzumab–paclitaxel dual therapy in this patient population [146]. Interestingly, while the mTOR inhibitor everolimus failed to significantly improve PFS in the overall cohort of pretreated HER2‐positive BC patients when combined with trastuzumab and chemotherapy (vinorelbine), it demonstrated a clinically meaningful 7.2‐month PFS prolongation specifically in the HR‐negative, HER2‐positive BC subgroup [147]. These findings collectively highlight both the therapeutic potential and molecular complexity of targeting the PI3K/AKT/mTOR pathway to overcome trastuzumab resistance while underscoring the importance of biomarker‐driven patient selection for optimal treatment outcomes.

### 3.4 Other Investigational Pathways

Beyond ADCs and TKIs, emerging pathways are being explored to address unmet needs in HER2‐positive BC, particularly for patients with refractory disease or rare molecular alterations.

#### 3.4.1 Farnesyl Transferase Inhibitors

Farnesyl transferase inhibitors (FTIs) were originally developed to target the posttranslational modification of RAS proteins by preventing their farnesylation, a critical process for membrane localization and subsequent activation of these oncogenic signaling molecules [148]. In HER2‐positive BC, HER2 overexpression drives RAS pathway hyperactivation, making FTIs a rational target [148]. Lonafarnib is a selective FTI that has shown promise in combination with standard anti‐HER2 therapy. A Phase I/II trial evaluated lonafarnib + trastuzumab + paclitaxel in HER2‐positive advanced BC, achieving a 58% ORR and median PFS of 14.5 months—superior to historical data for trastuzumab + paclitaxel alone [149]. While FTIs have not yet received regulatory approval for HER2‐positive BC, these results support further investigation in RAS‐activated subsets.

#### 3.4.2 HER3‐Targeted Antibodies

Beyond HER3‐targeted ADCs, HER3‐targeted antibodies are being developed to block ligand binding and prevent HER2‐HER3 dimerization. Seribantumab (a ligand‐blocking anti‐HER3 antibody) + trastuzumab showed a 17% ORR in trastuzumab‐resistant HER2‐positive BC (Phase II trial), with greater activity in patients with high HER3 ligand expression [150]. While this efficacy is modest, it highlights HER3 as a viable target for combination therapy.

The therapeutic landscape for HER2‐positive BC has advanced dramatically, with next‐generation ADCs such as T‐DXd redefining treatment for both HER2‐positive and HER2‐low disease. Novel ADCs targeting alternative antigens (e.g., HER3) and rational combinations to overcome resistance (e.g., anti‐HER2 agents + PI3K/AKT/mTOR inhibitors) continue to expand the therapeutic arsenal. Despite these successes, intratumoral heterogeneity and acquired resistance remain barriers, emphasizing the need for continued innovation to further improve long‐term outcomes for HER2‐positive BC patients.

## 4 Targeting Therapies for TNBC

TNBC represents 15%–20% of all BC cases and is recognized as one of the most aggressive subtypes with particularly poor clinical outcomes [9]. Defined by the absence of ER, PR, and HER2 expression, TNBC was historically deemed “nontargetable,” with chemotherapy as the sole systemic treatment option [10, 151]. However, advances in molecular profiling have uncovered significant intra‐subtype heterogeneity, paving the way for precision medicine strategies tailored to distinct biological drivers. This section reviews the evolution of TNBC targeting, from subtype classification to novel agent development.

### 4.1 Redefining TNBC: From “Nontargetable” to Subtype‐Specific Targeting

The paradigm shifts in TNBC management began with the recognition of its molecular heterogeneity. Molecular profiling studies have identified six distinct TNBC subtypes based on gene expression patterns and functional ontology [152, 153]:

Basal‐like 1 (BL‐1): High expression of proliferation markers and EGFR, sensitive to DNA‐damaging agents. Basal‐like 2 (BL‐2): Activation of the PI3K/mTOR pathway, responsive to PI3K inhibitors. Mesenchymal (M): Enrichment of stromal markers and potential sensitivity to antiangiogenic therapy. IM: High PD‐L1 expression and tumor‐infiltrating lymphocytes (TILs) responsive to immunotherapy. MSL: Stem cell‐like features under investigation for targeted stem cell therapies. LAR: AR expression, sensitive to AR antagonists.

**FIGURE 5:**
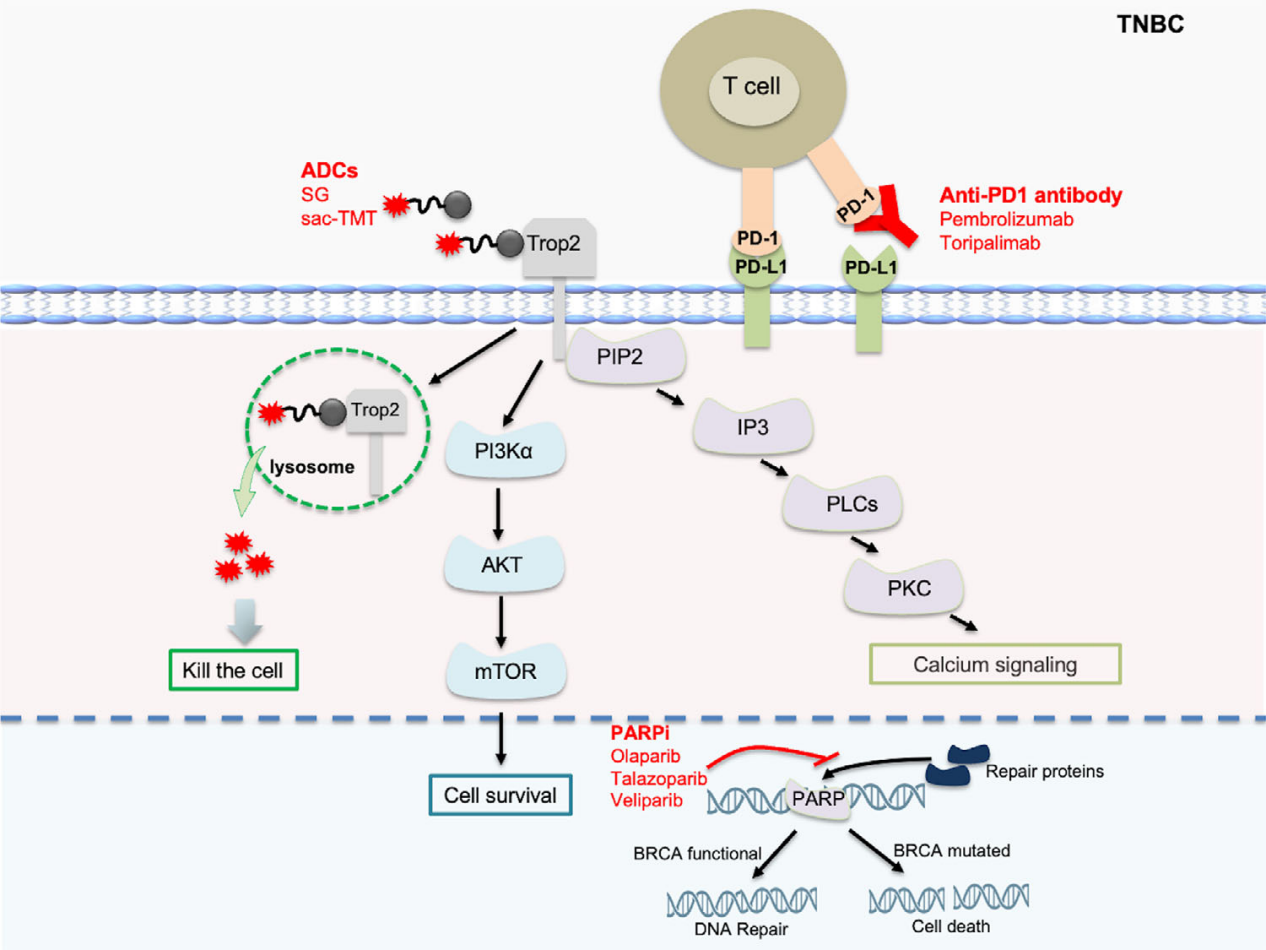
Targeted and immunotherapeutic strategies for triple‐negative breast cancer (TNBC). Highlights tailored therapeutic approaches for specific triple‐negative breast cancer (TNBC) subtypes, including PARP inhibitors (PARPi), trophoblast cell‐surface antigen 2‐directed antibody‒drug conjugates (Trop‐2 ADCs), and programmed death‐ligand 1 inhibitors (PD‐L1i).

This classification has transformed TNBC from a homogeneous “targetless” disease into distinct, subtype‐specific entities, thereby informing the development and approval of several tailored targeted therapies (Figure 5; Table 3). The identification of actionable biomarkers (e.g., *BRCA* mutations, PD‐L1 expression, AR status) further enables patient stratification, representing a pivotal step toward precision oncology in TNBC [154].

**TABLE 3:** Main clinically approved targeted agents for triple‐negative breast cancer (TNBC) treatment (as of 2025).

Agent	Clinical development (patient populations)	Clinical trials and efficacy	References
Olaparib (PARPi)	For patients with deleterious or suspected deleterious germline *BRCA*‐mutated (gBRCAm), HER2‐negative metastatic breast cancer	*OlympiAD*: Olaparib vs. physician's choice of chemotherapy (PFS: 7.0–4.2 months)	[155]
	For the adjuvant treatment of patients with deleterious or suspected deleterious gBRCAm, HER2‐negative high‐risk early breast cancer who have been treated with neoadjuvant or adjuvant chemotherapy	*OlympiA*: Olaparib vs. placebo: (3‐year iDFS: 85.9% vs. 77.1%)	[156]
Talazoparib (PARPi)	For patients with deleterious or suspected deleterious gBRCAm, HER2‐negative locally advanced or metastatic breast cancer	*EMBRACA*: Talazoparib vs. physician's choice of chemotherapy (PFS: 8.6 vs. 5.6 months)	[157]
Fuzuloparib (PARPi)	For patients with deleterious or suspected deleterious germline *BRCA*‐mutated (gBRCAm), HER2‐negative metastatic breast cancer, monotherapy or in combination with apatinib	*FABULOUS*: Fuzuloparib + apatinib vs. fuzuloparib vs. physician's choice of chemotherapy (PFS: 11.0 vs. 6.7 vs. 3.0 months)	[158]
Sacituzumab govitecan (SG) (Trop‐2 ADC)	For patients with unresectable locally advanced or metastatic triple‐negative breast cancer who have received two or more prior systemic therapies	*ASCENT*: SG vs. physician's choice of single‐agent chemotherapy (PFS: 5.6 vs. 1.7 months; OS: 12.1 vs. 6.7 months)	[159]
Sacituzumab tirumotecan (Sac‐TMT, SKB264)	For patients with unresectable locally advanced or metastatic triple‐negative breast cancer who have received two or more prior systemic therapies	*OptiTROP‐Breast01*: sac‐TMT vs. physician's choice of single‐agent chemotherapy (PFS: 6.7 vs. 2.5 months)	[160]
(Trop‐2 ADC)	For patients with locally recurrent, unresectable, or metastatic triple‐negative breast cancer whose tumors express PD‐L1 (CPS ≥ 10) in combination with chemotherapy	*KEYNOTE‐355*: Pembrolizumab + chemotherapy vs. placebo + chemotherapy (PFS: 9.7 vs. 5.6 months)	[161]
Pembrolizumab (PD‐L1i)	For high‐risk, early‐stage, triple‐negative breast cancer in combination with chemotherapy as neoadjuvant treatment, and then continued as a single agent as adjuvant treatment after surgery	*KEYNOTE‐522*: Pembrolizumab + chemotherapy vs. placebo + chemotherapy (pCR rate: 63% vs. 56%; 60‐month OS: 86.6% vs. 81.7%)	[162]
Toripalimab (PD‐L1i)	For the treatment of patients with untreated metastatic or recurrent PD‐L1‐positive triple‐negative breast cancer in combination with albumin‐bound paclitaxel	*TORCHLIGHT*: Toripalimab + nab‐paclitaxel vs. placebo + nab‐paclitaxel (PFS: 8.4 vs. 5.6 months; OS: 32.8 vs. 19.5 months)	[163]

### 4.2 Targeting DNA Repair Deficiency: PARPis for *BRCA*‐Mutant Cancers

Approximately 20% of TNBC patients harbor germline or somatic *BRCA1*/*2* mutations, leading to homologous recombination deficiency (HRD)—a vulnerability exploitable by PARPis [164, 165, 166]. PARP enzymes play a key role in repairing single‐strand DNA breaks (SSBs); inhibition of PARP in HRD tumors leads to unresolved SSBs, subsequent double‐strand breaks (DSBs), and synthetic lethality [167]. Preclinical studies have confirmed that TNBC is more sensitive to PARPis than non‐TNBC subtypes [168]. Subsequent clinical trials have demonstrated their survival benefits.

#### 4.2.1 Approved PARPis in TNBC

##### 4.2.1.1 Olaparib

The first PARPi approved for TNBC, based on the Phase III OlympiAD trial, where olaparib improved median PFS from 4.2 to 7.0 months compared to standard chemotherapy in *BRCA*‐mutated HER2‐negative metastatic BC (40% of enrolled patients were TNBC) [155]. The phase III OlympiA trial further expanded its use to the adjuvant setting, showing an 8.8% improvement in 3‐year DFS (85.9% vs. 77.1%) in *BRCA*‐mutated early TNBC [156], along with an OS benefit of 3.5 years (89.8% vs. 86.4%) [169].

##### 4.2.1.2 Talazoparib

Approved by the FDA in 2018, talazoparib exhibits strong PARP‐trapping activity, leading to superior efficacy compared to chemotherapy in the Phase III EMBRACA trial (median PFS: 8.6 vs. 5.6 months) [157]. However, no significant OS benefit was observed, a finding that may have been influenced by subsequent therapies [170].

##### 4.2.1.3 Fuzuloparib

A small‐molecule, orally active PARPi, fuzuloparib, was recently approved by the NMPA in 2025 based on the Phase III FABULOUS trial. The study demonstrated that fuzuloparib, both as monotherapy and in combination with apatinib, provided statistically significant improvements in PFS compared with chemotherapy in patients with HER2‐negative metastatic BC harboring germline *BRCA1*/*2* mutations. Specifically, the median PFS was 11.0 months for fuzuloparib plus apatinib, 6.7 months for fuzuloparib alone, and 3.0 months for the physician's choice of chemotherapy [158].

#### 4.2.2 Other PARPis and Biomarkers

##### 4.2.2.1 Veliparib

A nontrapping PARPi with reduced myelosuppression [171, 172], veliparib combined with carboplatin/paclitaxel, improved median PFS (14.5 vs. 12.6 months) in *BRCA*‐mutated advanced BC in the Phase III BROCADE3 trial [173], although this did not translate into an OS benefit [174].

##### 4.2.2.2 HRD Signatures

Beyond *BRCA* mutations, HRD scores (combining loss of heterozygosity, telomeric allelic imbalance, and large‐scale state transitions) predict PARPi response in BRCA‐wild‐type TNBC, expanding the patient population eligible for this therapy [175].

### 4.3 ADCs: Trop‐2 and Beyond

ADCs have emerged as a transformative class of agents for TNBC, leveraging tumor‐specific antigens to deliver potent cytotoxic payloads. Trop‐2 is highly expressed across all subtypes of BC and has been associated with tumor growth and poorer prognosis [176]. Its expression is particularly elevated in TNBC compared to other subtypes, underscoring its potential as a therapeutic target [177].

#### 4.3.1 Trop‐2‐Targeted ADCs

##### 4.3.1.1 Sacituzumab Govitecan

Approved by the FDA in 2020 for metastatic TNBC after ≥2 lines of therapy, sacituzumab govitecan (SG) consists of an anti‐Trop‐2 antibody conjugated to SN‐38 (a topoisomerase I inhibitor). The Phase III ASCENT trial showed that SG improved median PFS (5.6 vs. 1.7 months) and OS (12.1 vs. 6.7 months) compared to single‐agent chemotherapy [159]. SG also exhibits activity in early‐stage TNBC: the neoadjuvant Phase II NeoSTAR trial reported a pCR rate of 44% when combined with chemotherapy [178]. Notably, its benefits extend to HR‐positive BC, where it reduces the risk of progression or death in heavily pretreated patients [179].

##### 4.3.1.2 Datopotamab Deruxtecan (Dato‐DXd)

Dato‐DXd comprises a mAb linked to a topoisomerase inhibitor (deruxtecan), allowing precise delivery of its chemotherapeutic payload to TROP‐2‐expressing cancer cells through a 4:1 DAR. There is an ongoing Phase III TROPION‐Breast05 trial that aims to demonstrate the superiority of Dato‐DXd in combination with durvalumab (an anti‐PD‐L1 antibody) versus standard of care treatment in patients with PD‐L1‐high locally recurrent inoperable or metastatic TNBC [180].

##### 4.3.1.3 Sacituzumab Tirumotecan (Sac‐TMT, SKB264)

A next‐generation Trop‐2 ADC with a novel linker‐payload system (DAR = 7.4), Sac‐TMT, significantly prolonged PFS (6.7 vs. 2.5 months) in pretreated metastatic TNBC in the Chinese Phase III OptiTROP‐Breast01 trial, leading to NMPA approval in 2024 [160].

#### 4.3.2 Other ADC Targets in TNBC

##### 4.3.2.1 Glycoprotein NMB (gpNMB)

A transmembrane protein overexpressed in approximately 40% of TNBC cases plays a significant role in promoting tumor invasion and metastasis [181]. Glembatumumab vedotin (anti‐gpNMB ADC) improved median PFS (4.0 vs. 1.8 months) and OS (10.2 vs. 5.5 months) in advanced gpNMB‐positive TNBC in the Phase II EMERGE trial [182]. However, this initial promise was not confirmed in a subsequent randomized multicenter trial, which showed no PFS benefit (2.9 vs. 2.8 months) in a broader population of metastatic gpNMB‐expressing TNBC [183].

##### 4.3.2.2 Other Targets

The landscape of emerging ADC targets also encompasses numerous other molecules, notable examples being protein kinase PTK7, folate receptor alpha (FRα), B7‐H3 (CD276), CD25, CD166, CD205, nectin‐4, receptor tyrosine kinase‐like orphan receptors 1 and 2 (ROR1/ROR2), and mesothelin; these are currently under investigation in clinical trials and have been highlighted in recent scholarly reviews [184].

### 4.4 Harnessing the Immune System: Immunotherapy for PD‐L1‐Positive and Beyond

TNBC's high immunogenicity—characterized by elevated PD‐L1 expression and TIL infiltration—makes it uniquely responsive to immune checkpoint inhibition [185, 186]. Immunotherapy has evolved from monotherapy to combination strategies, with PD‐1/PD‐L1 inhibitors as the cornerstone.

#### 4.4.1 PD‐1/PD‐L1 Inhibitors

##### 4.4.1.1 Pembrolizumab

Approved by the FDA in 2020 for PD‐L1‐positive (combined positive score [CPS] ≥ 10) metastatic TNBC, pembrolizumab combined with chemotherapy improved median PFS (9.7 vs. 5.6 months) in the Phase III KEYNOTE‐355 trial [161]. In the neoadjuvant setting, the Phase III KEYNOTE‐522 trial demonstrated a pCR rate of 63% in patients receiving pembrolizumab in combination with chemotherapy, compared to 56% in those receiving chemotherapy alone [162].

##### 4.4.1.2 Atezolizumab

Mixed results were observed across clinical trials with atezolizumab. The Phase 1b trial reported a 39.4% ORR for atezolizumab plus nab‐paclitaxel in metastatic TNBC [187]. The subsequent IMpassion130 Phase III trial confirmed a PFS benefit with this combination in PD‐L1‐positive patients [188], while the IMpassion031 trial demonstrated improved pCR rates in early TNBC [189]. However, the IMpassion131 trial failed to show PFS or OS benefit with atezolizumab plus paclitaxel [190], leading to withdrawal of its indication for PD‐L1‐positive metastatic TNBC.

##### 4.4.1.3 Toripalimab

Approved by the NMPA in 2024 for PD‐L1‐positive (CPS ≥ 1) metastatic TNBC, toripalimab + nab‐paclitaxel improved median PFS (8.4 vs. 5.6 months) and OS (32.8 vs. 19.5 months) in the Phase III TORCHLIGHT trial [163].

#### 4.4.2 CTLA‐4 Inhibitors and Combination Strategies

##### 4.4.2.1 CTLA‐4 Blockade

Ipilimumab (anti‐CTLA‐4) combined with nivolumab (anti‐PD‐1) is under investigation in Phase II trials for metastatic TNBC, with preliminary data showing improved response rates in PD‐L1‐positive patients [191]. Tremelimumab (CTLA‐4 inhibitor) + durvalumab (PD‐L1 inhibitor) demonstrated enhanced clinical benefit in metastatic TNBC compared to ER‐positive BC in a Phase II trial [192].

##### 4.4.2.2 Bispecific Antibodies

KN046 (PD‐L1/CTLA‐4 bispecific antibody) + nab‐paclitaxel showed promising efficacy in first‐line metastatic TNBC, with a 50% ORR in PD‐L1‐positive patients and manageable toxicity [193].

#### 4.4.3 CAR T‐Cell Therapy

CAR T‐cell therapy, a revolutionary form of adoptive cell therapy, involves genetically modifying host T cells to express tumor‐specific receptors that enhance antitumor responses and has demonstrated remarkable clinical efficacy and durable responses in certain cancers [194]. While still in preclinical development for BC, promising approaches have emerged targeting TNBC. Zhou et al. developed a CAR T‐cell therapy targeting mucin 1 (MUC1), an antigen expressed in over 95% of TNBC cases, by engineering human T cells with a MUC28z CAR that specifically recognizes aberrantly glycosylated MUC1 in BCs. This approach demonstrated both target‐specific cytotoxicity and significant suppression of TNBC growth in xenograft models [195]. Similarly, recognizing EGFR as another promising target due to its frequent overexpression in TNBC, Xia et al. designed an EGFR‐targeted CAR T‐cell therapy that exhibited potent antitumor activity against TNBC cells while showing limited toxicity to normal tissues in both in vitro and in vivo studies [196]. These preclinical findings highlight the potential of CAR T‐cell therapy as a future treatment modality for TNBC, although further research is needed to translate these approaches into clinical applications.

### 4.5 Targeting the Tumor Microenvironment: Antiangiogenesis (VEGF)

VEGF, a key regulator of angiogenesis, demonstrates significantly higher intratumoral expression in TNBC than in non‐TNBC subtypes [197]. This biological characteristic makes VEGF an attractive therapeutic target, with inhibition strategies aimed at suppressing tumor neovascularization and preventing metastatic spread. The anti‐VEGF mAb bevacizumab has been extensively evaluated in clinical trials:

*Metastatic setting*: In a Phase III trial (RIBBON‐1), bevacizumab in combination with capecitabine or with a taxane/anthracycline regimen significantly prolonged median PFS. Specifically, PFS increased from 5.7 to 8.6 months in the capecitabine cohort and from 8.0 to 9.2 months in the taxane/anthracycline cohort. However, these regimens did not translate into a statistically significant improvement in OS [198, 199].

*Neoadjuvant setting*: The Phase III clinical trial demonstrated that adding bevacizumab to neoadjuvant chemotherapy increased pCR rates in HER2‐negative operable BC, including TNBC [200].

*Adjuvant setting*: The Phase III BEATRICE trial demonstrated that adding bevacizumab to chemotherapy did not improve 3‐year iDFS in patients with TNBC. Therefore, bevacizumab is not recommended as adjuvant therapy for unselected individuals in this population [201].

Limitations of bevacizumab include hypertension, bleeding, and proteinuria, restricting its long‐term use. Novel antiangiogenic agents (e.g., TKIs targeting VEGF receptors) are under investigation to improve efficacy and reduce toxicity [202].

### 4.6 Niche Targets: AR, EGFR, gpNMB

For specific TNBC subtypes, niche targets address unmet needs, particularly in patients with refractory disease.

#### 4.6.1 AR

Approximately 15%–50% of TNBC cases express AR, with the LAR subtype being AR‐dependent [203, 204], suggesting that AR‐targeted therapies may represent viable treatment options, particularly for patients with AR‐dependent TNBC [205]. Clinical evidence supports this therapeutic potential.

##### 4.6.1.1 Enzalutamide

A Phase II trial evaluating the AR inhibitor enzalutamide in patients with locally advanced or metastatic AR‐positive TNBC demonstrated both favorable tolerability and significant clinical activity, underscoring its promise as a targeted therapy for this subset [206]. Furthermore, the application of enzalutamide has been extended to the neoadjuvant setting. A recent Phase II trial indicated that the combination of enzalutamide and paclitaxel is effective in treating AR‐positive TNBCs that exhibited an insufficient response to AC (doxorubicin/cyclophosphamide) chemotherapy in the neoadjuvant context [207].

##### 4.6.1.2 Enobosarm

The combination of the selective AR modulator enobosarm with the immune checkpoint inhibitor pembrolizumab resulted in a modest 16‐week clinical benefit rate of 25% in heavily pretreated AR‐positive TNBC patients, without preselecting for PD‐L1 expression [208].

#### 4.6.2 EGFR

EGFR is frequently overexpressed in TNBC cases, particularly the BL‐1 subtype [209], making it a potential therapeutic target.

##### 4.6.2.1 Cetuximab

Phase II clinical trials evaluating the efficacy of cetuximab, an anti‐EGFR mAb, in metastatic TNBC demonstrated that combining cetuximab with cisplatin yielded a modest ORR (20%) and significantly improved median PFS and OS compared to cisplatin alone [210, 211]. However, despite these promising results, the overall clinical benefit remains limited, highlighting the need for further research to identify specific TNBC subpopulations that may exhibit greater sensitivity to EGFR inhibitors [212]. Such stratification could enhance treatment precision and improve outcomes for patients with this aggressive BC subtype.

##### 4.6.2.2 TKIs

TKIs (e.g., gefitinib and erlotinib) have shown minimal activity as monotherapy but may enhance the efficacy of chemotherapy in EGFR‐overexpressing TNBC [213].

TNBC has transitioned from a “targetless” disease to a subtype with tailored therapies, including Trop‐2 ADCs, PARPis for *BRCA*‐mutated cases, and immunotherapies for PD‐L1‐positive tumors. Emerging approaches such as CAR T‐cell therapy further expand treatment options for specific TNBC subsets. While significant progress has been made, TNBC remains associated with poor prognosis, highlighting the need for continued exploration of novel targets and combination strategies to address its aggressiveness and heterogeneity, building on the cross‐subtype platforms discussed in the following section.

## 5 Cross‐Subtype Therapeutic Platforms and Future Directions

BC treatment has long been guided by molecular subtype‐specific strategies, with therapies tailored to luminal (HR‐positive), HER2‐positive, and TNBC biology. However, recent advances have uncovered cross‐subtype therapeutic platforms—technologies or pathways that transcend subtype boundaries—offering opportunities to unify treatment approaches and address unmet needs across the disease spectrum [214, 215]. This chapter focuses on three such transformative platforms: ADCs as a modular technology, the PI3K/Akt/mTOR pathway as a ubiquitous oncogenic driver, and emerging modalities with potential across all BC subtypes.

### 5.1 The ADC Revolution: Reshaping the Treatment Landscape

The therapeutic paradigm for BC has been fundamentally transformed by the advent of ADCs. By combining the precise targeting of mAbs with the potent cytotoxicity of chemotherapeutic payloads, ADCs embody a versatile platform technology that transcends traditional subtype classifications based solely on HR and HER2 status [216, 217]. This “biological missile” strategy allows for the selective delivery of toxins to tumor cells, thereby maximizing efficacy while aiming to minimize systemic toxicity [218]. Over three decades of development have culminated in a new era of targeted cancer therapy, with ADCs now serving as crucial adjuvant, salvage, and metastatic treatments across the spectrum of BC [216]. The power of the ADC platform is best illustrated by its differential application across BC subtypes, primarily dictated by target antigen expression.

#### 5.1.1 HER2 as a Pan‐Subtype Target

Once considered relevant only to HER2‐positive disease, HER2‐targeted ADCs have redefined therapeutic boundaries. The second‐generation ADC T‐DM1 established the efficacy of this approach in HER2‐positive advanced BC [218]. The revolutionary step came with third‐generation ADCs such as T‐DXd. T‐DXd, with its potent topoisomerase I inhibitor payload, cleavable linker, and pronounced “bystander effect,” has demonstrated remarkable activity not only in HER2‐positive disease but also in tumors with low HER2 expression (IHC 1+ or 2+/ISH‐negative) [214]. This has effectively created a new, actionable “HER2‐low/‐ultralow” category, benefiting a significant portion of both HR‐positive and TNBC patients.

#### 5.1.2 Trop‐2 and the TNBC Frontier

Trop‐2 is expressed at low levels on the surface of normal epithelial cells and is overexpressed in BC, particularly in TNBC. Trop‐2‐targeted ADC, SG and Dato‐DXd have received FDA approval for the treatment of unresectable locally advanced or metastatic HR‐positive, HER2‐negative BC in patients who have received prior endocrine‐based therapy and at least two additional systemic therapies in the metastatic setting [77, 219]. For TNBC, a subtype historically lacking targeted options, the ADC platform has provided a critical breakthrough via the Trop‐2 antigen. SG and Dato‐DXd, which deliver the topoisomerase I inhibitor payload, have demonstrated significant improvements in metastatic TNBC [217]. This validates Trop‐2 as a high‐value target in this aggressive subtype and establishes ADCs as a cornerstone of its treatment.

#### 5.1.3 Beyond HER2 and Trop‐2

The clinical success of HER2 and Trop‐2 ADCs has catalyzed the exploration of novel targets to overcome resistance and expand treatable populations. A vast pipeline of over 200 investigational ADCs is underway, exploring antigens such as HER3, LIV‐1, and FRα, among others [214, 218]. Furthermore, the ADC platform itself is being engineered into next‐generation constructs, including bispecific ADCs (targeting two tumor antigens or an antigen and an immune cell receptor), ADCs with dual payloads, immune‐modulating ADCs, and radiolabeled drug conjugates [214].

However, the ADC revolution faces its own challenges. The emergence of resistance—through antigen downregulation, altered intracellular trafficking, or payload efflux—necessitates biologically informed sequencing strategies as patients become eligible for multiple ADCs. Furthermore, the expression of target antigens such as HER2 and Trop‐2 in some normal tissues raises concerns about on‐target, off‐tumor toxicity, which requires careful management [217]. Future progress hinges on refining biomarker quantification, elucidating resistance mechanisms, and personalizing ADC selection and sequencing to maximize long‐term patient benefit [218].

### 5.2 Targeting the PI3K/Akt/mTOR Pathway: A Ubiquitous Player Across Subtypes

The PI3K/Akt/mTOR signaling cascade is a central regulator of cell growth, survival, proliferation, and metabolism, and its dysregulation is a ubiquitous driver in BC pathogenesis [142]. Abnormal activation of this pathway occurs in nearly 70% of all BCs, making it a compelling cross‐subtype therapeutic target [51]. However, the mechanisms of activation and clinical implications exhibit both common themes and subtype‐specific nuances, necessitating a unified yet nuanced understanding for effective targeting. Common activating mechanisms include (1) gain‐of‐function mutations in *PIK3CA*, the gene encoding the p110α catalytic subunit, which are among the most common genomic alterations in BC, and (2) loss or inactivation of the tumor suppressor PTEN, a negative regulator that dephosphorylates PIP3 to halt pathway signaling. (3) Activating mutations in *AKT* (e.g., *AKT1 E17K*) and upstream receptor tyrosine kinases (e.g., HER2, EGFR) [142]. Despite these shared mechanisms, the frequency and context of alterations vary across subtypes, influencing therapeutic strategy:

*HR‐positive/HER2‐negative BC*: This subtype harbors the highest frequency of *PIK3CA* mutations (up to 47% in luminal A tumors), often co‐occurring with ER signaling [220]. Consequently, the pathway becomes a key mediator of endocrine resistance. While the mTOR inhibitor everolimus is approved in this setting, PI3Kα‐specific inhibitors (e.g., alpelisib and inavolisib) and AKT inhibitors (e.g., capivasertib) are increasingly used for *PIK3CA*‐mutated, hormone‐resistant advanced disease.
*HER2‐positive BC*: PI3K pathway activation serves as a major mechanism of escape from anti‐HER2 therapies. Aberrant signaling—through events such as *PIK3CA* mutation or constitutive AKT activation—contributes to both primary and secondary resistance to HER2‐targeted treatments [221]. Thus, combining PI3K/Akt/mTOR inhibitors with anti‐HER2 agents represents a rational strategy to overcome or prevent resistance, although their efficacy and safety profiles warrant further investigation [222].
*TNBC*: Abnormal activation of the PI3K/Akt/mTOR pathway is also frequently observed in TNBC, particularly within certain molecular subsets [220, 223]. For instance, the LAR subtype of TNBC is often characterized by *PIK3CA* mutations and PTEN loss, rendering it uniquely sensitive to inhibitors targeting the PI3K/Akt pathway [223]. While several studies have suggested the potential efficacy of PI3K/Akt/mTOR inhibitors in TNBC, their overall therapeutic effectiveness and safety profiles still require further investigation [222].

The clinical challenge in targeting this ubiquitous pathway lies in its essential physiological roles, which lead to dose‐limiting toxicities (e.g., hyperglycemia, rash, and stomatitis) with broad‐spectrum inhibitors. Future directions include developing highly isoform‐selective inhibitors (e.g., PI3Kα‐specific), identifying predictive biomarkers beyond *PIK3CA* mutations, and rationally combining pathway inhibitors with other targeted therapies, ADCs, or endocrine therapy to achieve synergistic efficacy while managing toxicity.

### 5.3 Novel Modalities and Horizons

Beyond ADCs and signaling pathway inhibitors, the therapeutic landscape of BC continues to evolve with novel modalities that employ distinct mechanisms of action. These emerging platforms hold the potential to overcome persistent therapeutic challenges, including tumor heterogeneity, immunosuppressive microenvironments, and previously undruggable intracellular targets, positioning them as versatile strategies applicable across multiple BC subtypes.

#### 5.3.1 Bispecific and Multi‐Specific Engagers

Building on antibody technology, these molecules are engineered to bind two or more distinct antigens simultaneously. A groundbreaking example is the “Multimodal Targeting Chimera” (Multi‐TAC) platform [224]. This system uses bioorthogonal chemistry to create multifunctional molecules that can, for instance, recruit both T cells and dendritic cells directly to tumor cells by binding EGFR on the tumor, CD3 on T cells, and PD‐L1 on dendritic cells. This forced tripartite interaction has shown potent antitumor activity and immune memory induction in preclinical models, including patient‐derived tumor organoids, offering a strategy to actively remodel the tumor immune microenvironment [224].

#### 5.3.2 Cell Therapies and Cell–Drug Conjugates

Although CAR T‐cell therapies have achieved only modest success in solid tumors, innovative cell‐based approaches are under development. For instance, HER2‐targeted CAR T cells show activity against tumors that have developed resistance to antibody‐based therapies, even when both target the same epitope. This indicates that CAR T cells are capable of infiltrating the tumor microenvironment, thereby addressing a key limitation of conventional antibody drugs [225]. Similarly, CAR T cells directed against MUC1 (MUC28z) have exhibited specific cytotoxicity in preclinical models of MUC1‐positive TNBC [226]. Cell–drug conjugates (CDCs) constitute an emerging therapeutic platform in which living cells—such as T cells, NK cells, platelets, or stem cells—are employed either as delivery vehicles or as enhanced therapeutic entities [227]. For example, tumor‐specific T cells can be conjugated with cytokines (e.g., IL‐2) or drug payloads to enhance their targeting and cytotoxic efficacy. Alternatively, platelets can be armed with anti‐PD‐L1 antibodies to localize checkpoint inhibitors to surgical resection sites, offering a potential strategy to prevent tumor recurrence [227]. This strategy capitalizes on the innate homing properties and extended circulation half‐life of cellular carriers, enabling improved targeting and pharmacokinetic profiles.

#### 5.3.3 Radiolabeled Conjugates and Pretargeted Strategies

Radioligand therapy is gaining traction. This involves conjugating a radioactive isotope (e.g., lutetium‐177) to a tumor‐targeting molecule. To improve the therapeutic index, pretargeted radioimmunotherapy (PRIT) strategies are being developed [228]. In PRIT, a nonradioactive bispecific fusion protein (e.g., one binding both a tumor antigen, such as CD38, and a radionuclide chelator) is administered first to localize at the tumor. After clearance from the blood, a radioactive payload is given, which rapidly binds to the prelocalized agent, delivering high radiation doses to the tumor while minimizing exposure to healthy tissues [228].

#### 5.3.4 PROTACs and Other Degradation Technologies

PROTACs offer a paradigm‐shifting approach to target proteins previously considered “undruggable,” such as transcription factors. These bifunctional molecules recruit an E3 ubiquitin ligase to a target protein, tagging it for proteasomal degradation. In BC, oral SERDs such as elacestrant represent the clinical forerunner of this concept, effectively degrading ERα in HR‐positive disease [217]. Next‐generation PROTACs are being explored to degrade a wider range of oncogenic drivers. The strategic advantage lies in their event‐driven catalytic mechanism, which can overcome resistance to traditional inhibitors.

The convergence of these platforms with established therapies defines the future direction of BC treatment. Rational combinations—such as ADCs with immunotherapy, PI3K inhibitors with CDK4/6 inhibitors, or degradation technologies with cellular therapies—hold the key to overcoming resistance. Furthermore, the “multi‐TAC” concept exemplifies the trend toward multimodal, integrated therapies designed to coordinately attack the tumor and its microenvironment [224]. As the menu of therapeutic modalities expands, the central challenge evolves from target discovery to the intelligent selection and sequencing of platforms, guided by deep molecular profiling and dynamic biomarkers, to deliver truly personalized and durable remissions for all BC patients.

Cross‐subtype platforms such as ADCs and PI3K/Akt/mTOR inhibitors have demonstrated transformative efficacy across HR‐positive, HER2‐positive, and TNBC subtypes, while emerging modalities such as bispecific engagers, CAR T‐cell therapy, and PROTACs address persistent challenges such as tumor heterogeneity and undruggable targets. The future of BC treatment lies in the intelligent integration of these platforms with established therapies, guided by deep molecular profiling and dynamic biomarkers. This convergence will enable truly personalized, multimodal strategies that overcome resistance and improve durable remissions, setting the stage for addressing remaining challenges in BC targeted therapy.

## 6 Challenges and Future Perspectives

The field of BC targeted therapy has undergone a paradigm shift—from “one‐size‐fits‐all” chemotherapy to subtype‐specific precision medicine—driven by advances in molecular profiling and agent development. However, persistent challenges, including drug resistance, inadequate biomarkers, and toxicity, limit optimal outcomes for many patients.

### 6.1 Overcoming Drug Resistance: Mechanisms and Strategies

Drug resistance is a primary impediment to effective BC treatment, affecting both conventional chemotherapy and molecularly targeted therapies [19]. Mechanisms of resistance are multifaceted and include the activation of alternative signaling pathways, mutations in drug targets, increased drug efflux, alterations in drug metabolism, and the influence of the tumor microenvironment [19]. For instance, endocrine resistance is frequently driven by *ESR1* mutations, RB1 loss (which mediates resistance to CDK4/6 inhibitors), and hyperactivation of the PI3K/AKT pathway (enabling bypass of ER signaling) [229]. Resistance to anti‐HER2 agents can result from HER3 upregulation, PI3K/AKT pathway mutations, and cleavage of the HER2 ECD, producing constitutively active fragments [230]. Immunotherapy resistance has been associated with PD‐L1 downregulation and modulation of the tumor microenvironment [231, 232], whereas resistance to PARPis often arises from *BRCA* reversion mutations [233].

Given the limitations of monotherapy, combination therapy has emerged as a promising strategy to overcome or delay resistance by simultaneously targeting multiple pathways or employing agents with complementary mechanisms of action [234]. Examples include the following: in luminal BC, combining ER‐targeting agents with CDK4/6 and PI3K inhibitors (e.g., inavolisib + palbociclib + fulvestrant [61]); in HER2‐positive disease, using ADCs alongside TKIs (e.g., T‐DXd + tucatinib [89]; and in TNBC, pairing ADCs with PARPis (e.g., SG + rucaparib [235]). In addition, the use of ctDNA enables early detection of resistance mutations, allowing for timely adjustment of treatment strategies [236]. Next‐generation agents such as PROTACs (e.g., vepdegestrant for enhanced ER degradation [31]) and novel ADCs (e.g., HER3‐DXd [237]) offer new avenues to overcome mutation‐driven resistance through direct and efficient targeting of oncoproteins.

### 6.2 The Quest for Predictive Biomarkers and Patient Selection

The advancement of precision therapy in BC hinges on the identification of robust biomarkers to accurately select patients who will benefit from specific treatments. Although established biomarkers such as HER2/neu, ER/PR, and Ki‐67 are fundamental for molecular subtyping and guiding therapy, significant challenges remain in the standardization and validation of emerging biomarkers across different subtypes.

While predictive biomarkers such as PD‐L1 expression, microsatellite instability‐high (MSI‐H) status, and tumor mutational burden (TMB) are FDA‐approved in various contexts, their utility in BC is often limited by inconsistent predictive value and a lack of comprehensive validation [238]. The future of precision medicine, therefore, depends on discovering novel biomarkers and understanding their dynamic evolution during treatment. Promising avenues include profiling microRNAs for early detection, prognosis prediction, and treatment monitoring [239] and using liquid biopsies to track ctDNA for real‐time assessment of mutations and therapeutic response [236]. and investigating exosomes as multifunctional carriers of biomolecules for diagnosis, prognosis, and disease monitoring [240].

Several key challenges impede the clinical application of biomarkers:

*Variable biomarker definitions*: A lack of global standardization for positivity thresholds leads to inconsistent patient selection. For example, the KEYNOTE‐522 trial demonstrated that the benefits of chemoimmunotherapy were independent of PD‐L1 status, offering no support for its use as a predictive biomarker in that context [241].
*False positives/negatives*: Spatial heterogeneity, missed by single‐site tissue biopsies (e.g., HER2‐low subclones within HER2‐positive tumors [242]), and the limited sensitivity of ctDNA assays (with detection limits of approximately 0.1% variant allele frequency [243]) can lead to inaccurate results.
*Unvalidated emerging biomarkers*: Several promising biomarkers, such as genomic scar‐based HRD scores beyond *BRCA* mutations for predicting PARPi response in TNBC [244] and TIL counts as correlates of immunotherapy efficacy [245], currently lack regulatory approval and standardized clinical implementation. To overcome these hurdles, future efforts should focus on (1) Multi‐omics profiling: Integrating genomic, transcriptomic, and proteomic data to develop composite biomarkers with superior predictive power [246]. (2) Liquid biopsy standardization: Harmonizing ctDNA detection methodologies—including NGS panel design and variant calling algorithms—and establishing clinically validated cutoffs for actionable mutations [243].

### 6.3 Toxicity Management and Treatment Sequencing

While BC targeted therapies have markedly improved patient outcomes by reducing the widespread adverse effects associated with traditional chemotherapy, they introduce unique toxicities that necessitate diligent management and thoughtful treatment sequencing to optimize therapeutic impact while maintaining quality of life. Each class of targeted therapy comes with distinct toxicity profiles, varying by the molecular mechanism involved. For example, CDK4/6 inhibitors such as palbociclib cause neutropenia in as many as 79% of patients at Grade ≥ 3, making hematologic monitoring a cornerstone of management [247]. PI3K inhibitors such as alpelisib are commonly linked to hyperglycemia, affecting 65% of patients, which necessitates baseline and ongoing glucose monitoring to mitigate risks [248]. ADCs such as T‐DXd are associated with a significant incidence of interstitial lung disease, observed in approximately 15% of patients, underscoring the need for rigorous pulmonary monitoring during treatment [249]. SG induces neutropenia in 63% of cases at Grade ≥ 3 severity, often requiring dose adjustments or supportive care [250]. Immunotherapies and PD‐1/PD‐L1 inhibitors, such as pembrolizumab, can trigger immune‐related adverse events, including colitis (10%), thyroiditis (8%), and pneumonitis (3%), all requiring careful immune‐modulatory interventions [251]. Effective management strategies involve proactive monitoring, toxicity‐guided dosing adjustments, and rational sequencing of therapies to preserve efficacy while minimizing side effects [252].

The sequencing of BC treatments is critical for optimizing efficacy while mitigating cumulative toxicity and overcoming resistance. The therapy order is driven by biological subtype, tumor stage, and prior treatment exposures. Key principles include [3]: (1) Luminal A/B BCs: These HR‐positive malignancies typically follow a cascade starting with endocrine therapy and CDK4/6 inhibitors, progressing to PI3K/AKT inhibitors for resistant disease, and integrating chemotherapy as a final line of therapy. (2) HER2‐positive BC: Dual HER2 blockade with trastuzumab and pertuzumab is the preferred first‐line therapy for early‐stage disease, followed by ADCs such as T‐DXd and TKIs (e.g., lapatinib) in advanced settings. (3) TNBC: TNBC necessitates a foundation of immunotherapy combined with chemotherapy. ADCs, including SG, are then prioritized for progressive disease, while *BRCA*‐mutated cases benefit from PARPis such as olaparib.

Continued innovation in targeted therapies accompanied by advancements in biomarker discovery, including predictive genomics or metabolomic profiling, will refine toxicity management strategies and sequencing paradigms [253]. Integrated analysis of clinical data, immune responses, and therapeutic resistance mechanisms promises individualized treatment mapping for BC subtypes, reducing trial‐and‐error approaches. Further research, especially involving real‐world evidence and adaptive trial designs, will expedite these advancements for clinical application.

### 6.4 The Impact of Advanced Technologies

Technological innovations are transforming BC research and clinical practice, addressing unmet needs from drug discovery to patient stratification. Advanced technologies are pivotal in driving the precision medicine revolution in BC. Next‐generation sequencing (NGS) and initiatives such as The Cancer Genome Atlas (TCGA) provide extensive genomic data, allowing for the identification of targetable genetic alterations beyond ER and HER2 [254]. Single‐cell RNA sequencing (scRNA‐seq) has revolutionized the understanding of intratumoral heterogeneity, delineating distinct cellular subpopulations—such as HER2‐low clones in HER2‐positive disease—and thereby uncovering the complex clonal architecture of tumors [255]. These insights provide a basis for tailoring effective combination therapies.

The integration of multi‐omics analyses (genomic, epigenomic, tumor microenvironment landscaping) with advanced imaging techniques is fundamental for comprehensive patient evaluation and precision therapy assignment [256]. Patient‐derived organoids (PDOs) serve as cutting‐edge preclinical platforms that replicate the biological complexity and heterogeneity of tumors, retaining key histological, genomic, and microenvironmental characteristics of the parent tissue for drug screening and personalized therapy development [257].

Liquid biopsy, by analyzing circulating cell‐free DNA (cfDNA), offers a less invasive method for real‐time monitoring of treatment response and the emergence of resistance mechanisms [258]. Furthermore, nanotheranostics, which combine diagnostics and therapy at the nanoscale, promise personalized healthcare management by improving drug delivery, enabling advanced imaging, and facilitating precise diagnostics and therapy monitoring [259].

Artificial intelligence (AI) and machine learning are becoming integral to several domains: (1) Drug discovery: AI is shifting ADC development from low‐throughput, empirical approaches to data‐driven, modular engineering. Integration of AI with multi‐omics, imaging, and clinical data holds promise for personalized ADC therapy. ADC‐specific models such as ADCNet and Linker‐GPT illustrate the power of multimodal AI for predicting ADC activity, payload toxicity, and linker stability [260]. (2) Patient stratification and response prediction: For ADCs, this process increasingly incorporates complex molecular signatures reflecting target accessibility and internalization efficiency. AI models leveraging digital pathology and multi‐omics data are emerging. For instance, a model trained on HER2‐low BC patients identified a multifeature signature (PR status, Ki‐67, HER2 expression) that may improve patient selection for T‐DXd [261]. Similarly, machine learning integrating MRI radiomics with genomic data has been used to predict pathological complete response to neoadjuvant therapy [262]. AI models are also being developed to anticipate resistance mechanisms, such as an XGBoost model using cGAS–STING pathway features to predict the anti‐PD‐1/PD‐L1 response [263]. (3) Clinical decision support: Tools such as IBM Watson for Oncology can recommend subtype‐specific therapies, aiding treatment decision‐making, particularly in settings with limited expert resources [264].

## 7 Conclusions

The management of BC is advancing rapidly, fuelled by growing insights into its molecular complexity and the development of targeted treatments. Although substantial progress has been achieved—particularly in molecular subtyping and the use of advanced agents such as ADCs—significant challenges remain. These include therapy resistance and the need for more reliable predictive biomarkers [19]. Future efforts will likely focus on deciphering novel resistance mechanisms and designing strategies to overcome them, potentially through multidrug delivery platforms, nanocarriers, and innovative combination regimens [265]. The pursuit of highly accurate predictive biomarkers will also intensify, shifting toward dynamic monitoring via liquid biopsy and integration of multi‐omics data to enable truly personalized treatment selection [266]. In addition, optimizing treatment sequencing and managing toxicities will remain central to improving patient outcomes and quality of life.

Conquering this complex disease will require sustained collaboration among all stakeholders. The future of BC care lies in dynamic, personalized treatment strategies—enabled by real‐time biomarker assessment (e.g., ctDNA for minimal residual disease), multimodal combinations, and technology‐facilitated translation from bench to bedside (e.g., AI and PDOs). By addressing these priorities, we move closer to the goal of transforming BC from a heterogeneous disease into a manageable, and ultimately curable, condition.

## Author Contributions

**Aiyu Liu**: conceptualization, writing – original draft. **Puchao Peng**: conceptualization, writing – original draft. **Yeke Zhu**: writing – original draft. **Qiewen Fei**: writing – original draft. **Weiwei Liu**: conceptualization, writing – review and editing. **Shizhen Zhang**: conceptualization, writing – review and editing. All authors have read and approved the final manuscript.

## Funding

This study was supported by grants from the National Natural Science Foundation of China Project (Grant nos. 82473005 and 82202838).

## Ethics Statement

The authors have nothing to report.

## Conflicts of Interest

The authors declare no conflicts of interest.

## Data Availability

The authors have nothing to report.
